# Validation and refinement of the 2022 European LeukemiaNet genetic risk stratification of acute myeloid leukemia

**DOI:** 10.1038/s41375-023-01884-2

**Published:** 2023-04-11

**Authors:** Christian Rausch, Maja Rothenberg-Thurley, Annika Dufour, Stephanie Schneider, Hanna Gittinger, Cristina Sauerland, Dennis Görlich, Utz Krug, Wolfgang E. Berdel, Bernhard J. Woermann, Wolfgang Hiddemann, Jan Braess, Michael von Bergwelt-Baildon, Karsten Spiekermann, Tobias Herold, Klaus H. Metzeler

**Affiliations:** 1 Laboratory for Leukemia Diagnostics, Department of Medicine III, University Hospital, LMU Munich, Munich, Germany; 2 Institute of Human Genetics, University Hospital, LMU Munich, Munich, Germany; 3 Institute of Biostatistics and Clinical Research, University of Münster, Münster, Germany; 4 Department of Medicine 3, Klinikum Leverkusen, Leverkusen, Germany; 5 Department of Medicine A, University Hospital Münster, Münster, Germany; 6 German Society of Hematology and Oncology, Berlin, Germany; 7 German Cancer Consortium (DKTK), Partner Site Munich, Munich, Germany; 8 German Cancer Research Center (DKFZ), Heidelberg, Germany; 9 Department of Oncology and Hematology, Hospital Barmherzige Brüder, Regensburg, Germany; 10 Bavarian Cancer Research Center (BZKF), Munich, Germany; 11 Department of Hematology, Cellular Therapy and Hemostaseology, University Hospital Leipzig, Leipzig, Germany

**Keywords:** Risk factors, Cancer genomics, Acute myeloid leukaemia

## Abstract

The revised 2022 European LeukemiaNet (ELN) AML risk stratification system requires validation in large, homogeneously treated cohorts. We studied 1118 newly diagnosed AML patients (median age, 58 years; range, 18–86 years) who received cytarabine-based induction chemotherapy between 1999 and 2012 and compared ELN-2022 to the previous ELN-2017 risk classification. Key findings were validated in a cohort of 1160 mostly younger patients. ELN-2022 reclassified 15% of patients, 3% into more favorable, and 12% into more adverse risk groups. This was mainly driven by patients reclassified from intermediate- to adverse-risk based on additional myelodysplasia-related mutations being included as adverse-risk markers. These patients (*n* = 79) had significantly better outcomes than patients with other adverse-risk genotypes (5-year OS, 26% vs. 12%) and resembled the remaining intermediate-risk group. Overall, time-dependent ROC curves and Harrel’s C-index controlling for age, sex, and AML type (de novo vs. sAML/tAML) show slightly worse prognostic discrimination of ELN-2022 compared to ELN-2017 for OS. Further refinement of ELN-2022 without including additional genetic markers is possible, in particular by recognizing *TP53*-mutated patients with complex karyotypes as “very adverse”. In summary, the ELN-2022 risk classification identifies a larger group of adverse-risk patients at the cost of slightly reduced prognostic accuracy compared to ELN-2017.

## Background

In 2010, an expert panel on behalf of the European LeukemiaNet (ELN) developed guidelines for diagnosis and management of acute myeloid leukemia (AML) in adults [1]. In 2017, an updated version was published [2], including a risk stratification system based on cytogenetic and molecular aberrations. The ELN-2017 risk classification has been validated in intensively treated AML patient cohorts [3–9], and found widespread adoption in routine practice and clinical trials. In 2022, another update of the ELN guidelines has been published [10]. This latest version introduced multiple changes to the risk stratification system. First, patients with internal tandem duplications of *FLT3* (*FLT3*-ITD) in the absence of core binding factor (CBF) rearrangements or adverse-risk markers are now considered intermediate risk, regardless of *FLT3*-ITD-to-wild-type (wt) allelic ratio or *NPM1* co-mutation. This change is not only intended to account for the impact of *FLT3* inhibitors on outcomes, but also comes in the wake of several validation studies not showing different outcomes for patients with low vs. high *FLT3-*ITD allelic ratios [4, 11]. In contrast, we and others did confirm that consideration of *FLT3*-ITD allelic ratio improved risk stratification within ELN-2017 [3, 6, 9, 12]. Second, it has become clear that only in-frame mutations in the leucine zipper domain of *CEBPA* (*CEBPA*^bZIP-inf^) predict favorable outcomes [13], and consequently, only those are considered favorable-risk according to ELN-2022 – regardless of whether they occur alone or with a second *CEBPA* mutation. Finally, in the adverse-risk category, t(8;16)(p11;p13)/*KAT6A::CREBBP* and t(3q26.2;v)/*MECOM*(*EVI1*) rearrangements, and mutations in *BCOR, EZH2, SF3B1, SRSF2, STAG2, U2AF1* or *ZRSR2* in the absence of favorable-risk markers have been added as poor-risk markers.

While the proposed changes individually are supported by published data, the effects of these modifications on overall risk stratification have not yet been validated in large and homogeneously treated cohorts. We set out to test the prognostic relevance of the ELN-2022 classification in intensively treated AML patients, and to compare this revised risk stratification to the prior ELN-2017 system.

## Patients and methods

We studied 1138 newly diagnosed AML patients (median age, 58 years [y]; range, 18–86 y) who received cytarabine-based induction chemotherapy in two subsequent multicenter phase III trials of the German AML Cooperative Group (AMLCG-1999, clinicaltrials.gov identifier NCT00266136, *n* = 864; and AMLCG-2008, NCT01382147, *n* = 274) between 1999 and 2012 [14–17]. Treatment regimens are summarized in the Supplementary Methods, and patient disposition is detailed in a previous report [3]. None of the patients received *FLT3* inhibitors or gemtuzumab ozogamicin during first-line treatment. AML was diagnosed according to World Health Organization 2008 criteria [18]. Metaphase cytogenetics were analyzed centrally, and patients were profiled for mutations in 68 genes commonly mutated in myeloid neoplasms via targeted sequencing from bone marrow (BM) or peripheral blood (PB), as described previously [19]. The limit of detection was a variant allele frequency of ≥2%. Variants were classified in accordance with widely accepted consensus classifications [20, 21]. Twenty subjects were excluded due to missing genetic data. All study protocols were in accordance with the Declaration of Helsinki and approved by the institutional review boards of participating centers. All patients provided written informed consent for inclusion on the clinical trial and genetic analyses. Median follow-up of survivors was 98 months [22].

Key findings were validated in a published cohort of 1160 mostly younger AML patients (83% aged <60 y) treated with intensive induction chemotherapy on clinical trials of the Acute Myeloid Leukemia Study Group (AMLSG, Supp. Table 1) [23].

We studied associations between ELN genetic risk groups and other patient characteristics using Fisher’s exact test for categorical and the Wilcoxon rank-sum test for continuous variables. We used widely accepted definitions of common clinical endpoints (complete remission [CR], relapse-free survival [RFS], and overall survival [OS]) (Supplementary Methods) [24]. For time-to-event analyses, we calculated survival estimates using the Kaplan–Meier method except in the case of allogeneic stem cell transplant (alloSCT), where we used Simon–Makuch plots, as described in the Supplementary Methods. We compared groups by the log-rank test. We used multivariable logistic regression models to analyze factors associated with achievement of CR, and Cox proportional hazards models for survival endpoints. All multivariable models were stratified by trial arm to control for possible differences between cohorts. Potential models were tested using Akaike’s ‘An Information Criterion’. Collinearity of variables was tested using variance inflation factor. Statistical analyses were performed using R version 4.2.1 (R Foundation for Statistical Computing, Vienna, Austria).

## Results

### Association of the ELN-2022 risk groups with baseline demographics and comparison to ELN-2017

**Fig. 1:**
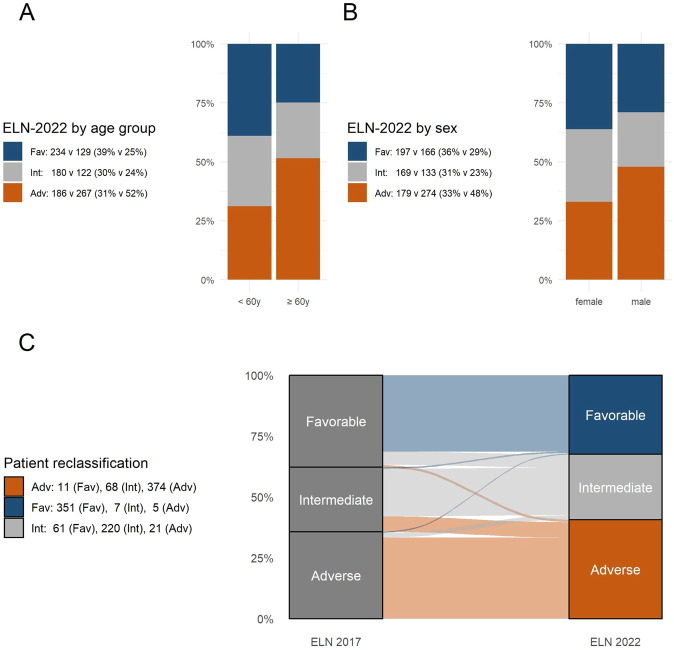
Patient distribution according to ELN-2017 and 2022. **A** ELN-2022 categories stratified by age group (<60 y vs. ≥60 y). **B** ELN 2022 classification stratified by sex. **C** ELN-2022 classification compared to ELN-2017.

Out of 1118 patients stratified according to ELN-2022, 363 (32%) were classified as favorable, 302 (27%) as intermediate, and 453 (41%) as adverse-risk (Table 1). For those <60 y of age (*n* = 600), the distribution was 39%, 30%, and 31%, compared to 25%, 24%, and 52% for those aged ≥60 y (*n* = 518) (Fig. 1A). Similar to ELN-2017, ELN-2022 adverse risk significantly associated with older age (*p* < 0.0001 for ELN favorable/intermediate vs. adverse), male sex (*p* = 0.003), secondary AML (sAML; *p* = 0.0006), and a lower white blood cell (WBC) count at diagnosis (*p* < 0.0001). These associations persisted in patients aged <60 y or ≥60 y. We did not find a significant association between ELN-2022 adverse risk and tAML (*p* = 0.21). The significantly higher proportion of adverse-risk genetics among male patients (48% vs. 33%, *p* < 0.0001, Fig. 1B) was largely due to a lower prevalence of *NPM1* mutations and higher prevalence of *RUNX1* and *ASXL1* mutations in males (*p* < 0.0001 and *p* = 0.009, respectively). In addition, some of the newly recognized adverse risk-defining mutations were also significantly associated with male sex (*EZH2*: *p* = 0.0002; *SRSF2*: *p* < 0,0001; *STAG2*: *p* = 0.0075; *U2AF1*: *p* = 0.0016; *ZRSR2*: *p* = 0.0005).

**Table 1:** Patient characteristics according to ELN-2022 risk groups.

	All patients	ELN-2022 risk group			*p*
		Favorable	Intermediate	Adverse	
Patients, *n*	1 118	363	302	453	–
Age, median (range)	58 (18–86)	52 (18–86)	55 (18–83)	62 (21–80)	<0.0001
Male sex	573	166	133	274	0.003
AML subtype					
De-novo AML	937	334	258	345	–
Secondary AML	124	17	27	80	0.0006
Therapy-related AML	57	12	17	28	0.2126
WBC at diagnosis [G/l], median (range)	20.4 (0.1–798)	24.1 (0.4–798)	36.5 (0.1–786)	11.5 (0.5–666)	<0.0001
Bone marrow blasts [%], median (range)	80 (6–100)	80 (6–100)	83 (10–100)	71 (9–100)	0.002
ELN-2017 risk group					
Favorable	423	351	61	11	–
Intermediate	295	7	220	68	–
Adverse	400	5	21	374	–
Gene Mutations					
*ASXL1*	135	14	2	119	<0.0001
*BCOR*	90	6	3	81	<0.0001
*CEBPA*^bZIP-inf^	44	44	0	0	0.0001
*CEBPA*^other^	39	8	18	13	0.4085
*DNMT3A*	391	137	157	97	0.0022
*EZH2*	44	11	1	32	0.0002
*FLT3-*ITD	303	14	204	85	0.0032
*FLT3-*ITD low	131	11	78	42	0.0374
*FLT3*-ITD high	172	3	126	43	0.0114
*IDH1*	98	43	29	26	0.0034
*IDH2*	165	43	48	74	0.2308
*KMT2A*-PTD^a^	56	1	17	38	0.0028
*KRAS*	73	26	19	28	0.7133
*NPM1*	431	239	164	28	<0.0001
*NRAS*	216	98	39	79	0.1902
*PTPN11*	114	59	18	37	0.0699
*RAD21*	54	27	17	10	0.0006
*RUNX1*	178	7	0	171	0.0087
*TET2*	192	65	51	76	0.8087
*TP53*	81	2	4	75	<0.0001
*SF3B1*	45	4	1	40	<0.0001
*SRSF2*	135	21	1	113	<0.0001
*STAG2*	98	17	1	80	0.0075
*U2AF1*	38	3	0	35	0.0016
*WT1*	143	48	43	52	0.2756
*ZRSR2*	12	2	0	10	0.0005

^a^KMT2A-PTD status was unknown in 374 patients.

Compared to ELN-2017, 85% of patients remained in the same risk group. This substantial agreement between ELN-2017 and ELN-2022 was confirmed by Cohen’s kappa (unweighted kappa: 0.73 (95% CI: 0.77–0.80), weighted kappa: 0.84 (95% CI: 0.87–0.89)). Fifteen percent of patients (*n* = 171; 14% of male and 17% of female patients) were classified into a different ELN-2022 risk category, with 3% moving into a more favorable and 12% into a less favorable category (Fig. 1C). Reasons for reclassification are detailed in Supplementary Table 2.

### Outcomes of AML patients classified according to the ELN-2022 risk stratification

**Fig. 2:**
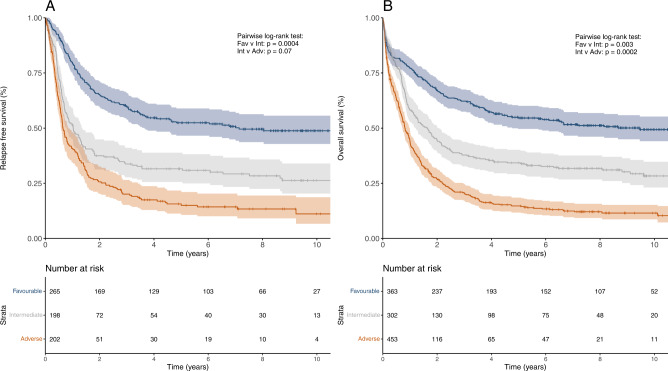
Outcomes of patients according to the ELN-2022 risk groups. **A** Relapse-free survival and **B** overall survival in the entire cohort of 1118 patients. (age range: 18–86).

CR rates for patients in the ELN-2022 favorable, intermediate, and adverse risk groups were 73%, 66%, and 45% (Table 2). For the corresponding ELN-2017 categories, CR rates were 72%, 66%, and 41% (Supplementary Table 3). Five-year RFS was 52%, 32%, and 16%, respectively, for the ELN-2022 risk groups compared to 53%, 26%, and 12% for the corresponding ELN-2017 groups. Median RFS by ELN-2022 category was 7.2 y (95% CI, 3.7y-not reached), 1.0 y (95% CI, 0.8–1.4 y) and 0.7 y (95% CI, 0.6–0.9 y) for the favorable, intermediate, and adverse group (Fig. 2A), compared to 7.1 y (95% CI, 3.7y-not reached), 1.0 y (95% CI, 0.7–1.3 y), and 0.6 y (95% CI, 0.6–0.8 y) for the corresponding ELN-2017 categories.

**Table 2:** Outcomes according to ELN-2022 risk groups.

ELN-2022 risk group	CR rate [%]	*p*	5 y RFS [%] (95% CI)	*p*	5 y OS [%] (95% CI)	*p*
All patients						
Favorable	73	<0.0001	52.4 (46.6–58.9)	<0.0001	54.6 (49.6–60.0)	<0.0001
Intermediate	66		31.5 (25.6–38.8)		34.2 (29.2–40.1)	
Adverse	45		15.6 (11.3–21.7)		14.8 (11.8–18.5)	
Patients < 60a						
Favorable	75	<0.0001	60.2(53.2–68.0)	0.0002	62.2 (56.2–68.8)	0.006
Intermediate	67		41.3 (33.4–51.2)		44.2 (37.4–52.2)	
Adverse	46		29.1 (20.9–40.7)		25.1 (19.5–32.3)	
Patients ≥ 60a						
Favorable	70	<0.0001	37.0 (28.1–48.8)	0.0002	40.6 (32.8–50.2)	0.008
Intermediate	64		16.1 (4.2–26.9)		19.4 (13.4–28.1)	
Adverse	43		5.6 (2.3–12.4)		7.6 (4.9–11.7)

Five-year OS by risk group was 55%, 34%, and 15% for ELN-2022 (Table 2), versus 54%, 31%, and 12% for the corresponding ELN-2017 categories. Median OS by ELN-2022 risk group was 9.5 y (95% confidence interval (CI), 4.8–12.3 y), 1.7 y (95% CI, 1.2–2.0 y), and 0.8 y (95% CI, 0.7–1.0 y) (Fig. 2B), compared to 8.2 y (95% CI, 4.6–11.9 y), 1.7 y (95% CI, 1.2–2.0 y), and 0.8 y (95% CI: 0.7–0.9 y) for the ELN-2017 categories. In analyses stratified by age (<60 y versus ≥60 y), the ELN-2022 classification maintained its prognostic impact in both age groups (Supplementary Fig. 1A–D).

**Fig. 3:**
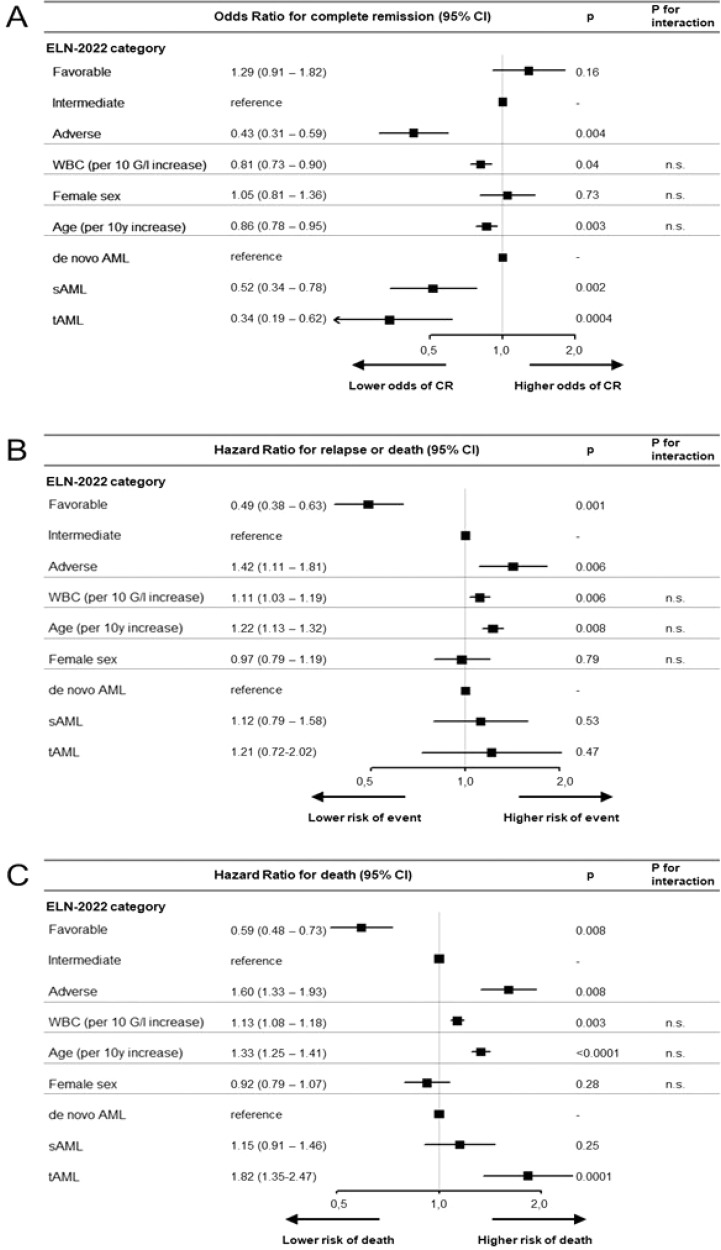
Multivariate analyses of outcomes according to the ELN-2022 genetic risk groups and further pretreatment prognostic variables. **A** Forest plot showing odds ratios from a logistic regression model for achievement of complete remission. **B** Forest plot showing hazard ratios from a Cox proportional hazards model for relapse-free survival. **C** Forest plot showing hazard ratios from a Cox proportional hazards model for overall survival. Interaction *P* values refer to an interaction between the ELN-2022 risk groups and the respective variable.

In a multivariate model assessing factors associated with achievement of CR, ELN-2022 adverse risk, older age, higher WBC count, and a diagnosis of sAML or tAML were significantly associated with a lower likelihood of attaining CR (Fig. 3A). In a multivariate model for RFS, ELN-2022 favorable risk associated with longer RFS, while ELN-2022 adverse risk, older age and higher WBC count associated with shorter RFS (Fig. 3B). Those same factors also associated with survival in a multivariate model for OS (Fig. 3C). In addition, tAML associated with shorter OS.

Receiver operating characteristic (ROC) curves showed numerically lower areas under the curves for the associations of ELN-2022 with OS and RFS, compared to ELN-2017. ELN-2022 performed significantly worse at some timepoints (Supplementary Fig. 2A, B). Harrel’s C-index confirmed a slightly lower prognostic accuracy of ELN-2022 for OS, with values of 0.658 for ELN-2022, and 0.664 for ELN-2017, when controlling for age, sex, and presence of sAML or tAML.

**Fig. 4:**
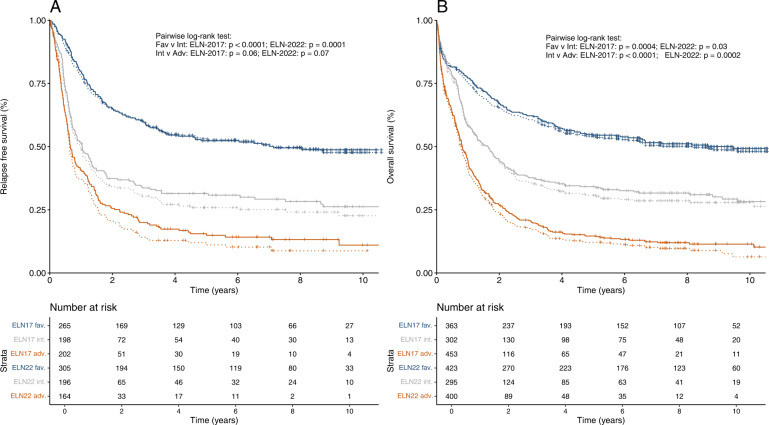
Outcomes of patients according to the ELN-2017 and ELN-2022 risk groups. **A** relapse-free survival, and **B** overall survival in the entire cohort of 1118 patients (age range: 18–86). Dashed lines represent ELN-2017 risk groups, solid lines represent ELN-2022 risk groups.

Kaplan–Meier plots for the entire cohort classified using either ELN-2022 or ELN-2017 are shown in Fig. 4. We observed a trend towards better outcomes for the ELN-2022 adverse group compared to ELN-2017 adverse-risk patients. We therefore analyzed outcomes of reclassified subgroups in more detail.

### Outcomes of reclassified subgroups

RFS and OS of reclassified patients are detailed in Supplementary Fig. 3. Only twelve patients were reclassified from the ELN-2017 adverse or intermediate groups into the ELN-2022 favorable risk-group, precluding formal outcome analyses for this subset (Supplementary Fig. 3A, B). The 61 patients reclassified from ELN-2017 favorable to ELN-2022 intermediate risk had a numerically higher 5 y OS rate than other ELN-2022 intermediate-risk patients (48% vs. 33%, Supplementary Fig. 3D; *p* = 0.307). In contrast, patients reclassified from ELN-2017 adverse to ELN-2022 intermediate risk (*n* = 21) had numerically worse 5 y OS than other intermediate risk patients (10% vs. 33%, Supplementary Fig. 3D; *p* = 0.068), and significantly worse OS than those reclassified from favorable to intermediate risk (*p* = 0.016). Finally, patients reclassified from ELN-2017 intermediate to ELN-2022 adverse risk (*n* = 68) achieved significantly better 5 y OS than other adverse-risk patients (25% vs. 12%, *p* = 0.007; Supplementary Fig. 3F).

### Outcomes stratified by postremission therapy

Of all patients in our cohort, 665 reached CR. Of those, 109 underwent alloSCT in first CR (CR1; 97 aged <60 y and 12 aged ≥60 y). Since alloSCT in CR1 was rare in older patients, we analyzed outcomes according to postremission therapy only in patients <60 y who achieved CR1. This subgroup (*n* = 381) is characterized in detail in Supplementary Table 4. Even though our cohort was largely recruited before the widespread adoption of risk scores incorporating molecular genetics, we found a significant association of alloSCT in CR1 with ELN-2022 risk group (18% of favorable, 30% of intermediate, and 34% of adverse risk patients, *p* < 0.0001).

Supplementary Fig. 4 shows RFS and OS of ELN-2022 favorable, intermediate, and adverse-risk patients stratified by postremission therapy. In proportional hazards models calculated within each risk group using transplant status as a time-dependent covariable, RFS was numerically better for those receiving allogeneic transplant compared with those receiving chemotherapy or ASCT within all groups. However, this difference was significant only for favorable and adverse risk patients (*p* = 0.0028 and *p* = 0.024, respectively; Supplementary Fig. 4A, E). In the ELN-2022 favorable and intermediate groups, OS was not significantly different between patients receiving or not receiving an allogeneic transplant in CR1, while in the adverse group, those receiving alloSCT in CR1 had significantly better OS (*p* = 0.032, Supplementary Fig. 4F). Overall, these findings mirror our previous results using the ELN-2017 risk categories, where adverse risk patients also were the only group having a significant OS benefit from alloSCT. However, since postremission therapy assignment was not randomized, these results are likely to be biased by factors other than baseline genetic risk that have influenced treatment decisions (e.g., comorbidities and performance status). In addition, improved transplant protocols with lower treatment-related mortality may shift this balance in favor of allogeneic transplantation [25].

### Outcomes of patients within genetic subsets of the ELN-2022 categories

Outcomes of specific subsets within the ELN-2022 risk categories are presented in detail in the Supplement (Supplementary Table 5, Supplementary Figs. 5, 6). The following observations seem particularly noteworthy: Due to the elimination of *FLT3*-ITD:wt allelic ratio from the classification, most patients with *FLT3*-ITD are now classified as intermediate-risk. In support of this modification, ELN-2022 intermediate-risk patients with *FLT3*-ITD had similar outcomes to other intermediate patients (5 y OS, 35% vs. 34%; Supplementary Fig. 5A, B). Next, we analyzed whether *FLT3*-ITD allelic ratio would add discriminatory power. While there was no significant OS difference between *FLT3*-ITD^high^ versus *FLT3*-ITD^low^ patients within the intermediate risk group, those with *FLT3*-ITD^low^ (*n* = 78; 5 y OS, 45%) tended to have better survival than *FLT3*-ITD^high^ patients (*n* = 126; 5 y OS, 27%) (Supplementary Fig. 5C, D, *p* = 0.097). Among patients with *FLT3*-ITD in the adverse-risk group, there was a significant difference between the *FLT3*-ITD^high^ and *FLT3*-ITD^low^ subgroups (ITD-high: *n* = 43; 5 y OS, 10%; ITD-low: *n* = 42; 5 y OS, 25%; *p* = 0.027; Supplementary Fig. 5C, D). *FLT3*-ITD mutations were too rare in the ELN-2022 favorable-risk group (*n* = 14) to allow similar comparisons.

### Outcomes of patients with myelodysplasia-related mutations

**Fig. 5:**
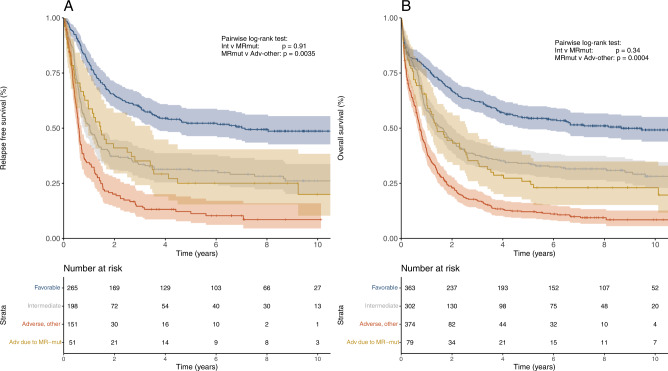
Outcomes of patients newly classified as adverse due to presence of a myelodysplasia-related mutation compared to ELN-2022 risk groups. **A** Relapse-free survival and **B** overall survival in the entire cohort of 1118 patients (age range: 18–86). The adverse risk group is divided into patients newly classified as adverse due to presence of a myelodysplasia-related mutation (gold) and all other adverse risk patients (orange).

Seventy-nine patients, or 45% of all re-classified patients, were moved from ELN-2017 favorable (*n* = 11) or intermediate (*n* = 68) to ELN-2022 adverse risk based on the inclusion of additional myelodysplasia-related (MR) mutations (*BCOR*, *EZH2*, *SF3B1*, *SRSF2*, *STAG2*, *U2AF1*, *ZRSR2*) as poor-risk markers. The presence of MR mutations significantly correlated with older age (46/79 reclassified patients were aged ≥60 y) and male sex (48/79) (*p* < 0.0001 for both). These re-classified patients had significantly better RFS (5 y RFS, 25% vs. 12%; median RFS, 1.5 y vs. 0.6 y; *p* = 0.0035) and OS (5 y OS, 26% vs. 12%; median OS, 1.7 y vs. 0.7 y; *p* = 0.0004) than patients with other adverse-risk genotypes (including *ASXL1*, *RUNX1* or *TP53* mutations*)*, and did not show a significant difference in RFS (*p* = 0.91) or OS (*p* = 0.34) compared to the ELN-2022 intermediate cohort (Fig. 5). Although patients with MR-associated mutations seemed to have a particularly strong benefit from alloSCT in CR1 (OS *p* = 0.0026; Supplementary Fig. 4G, H), the limited patient number (*n*_alloSCT_ = 6) precludes definitive conclusions.

These findings were confirmed in an independent validation cohort of 1160 patients. Here, we also found significantly better OS for those reassigned to adverse risk based on MR mutation positivity (5 y OS, 30% vs. 18%; median OS, 1.6 vs. 1.0 y; *p* = 0.0052) compared to other adverse risk patients. In the validation cohort, OS of reassigned MR-mutated patients was significantly worse than for the remaining intermediate-risk group (*p* = 0.02).

### Potential refinement of ELN-2022 without inclusion of additional markers

**Fig. 6:**
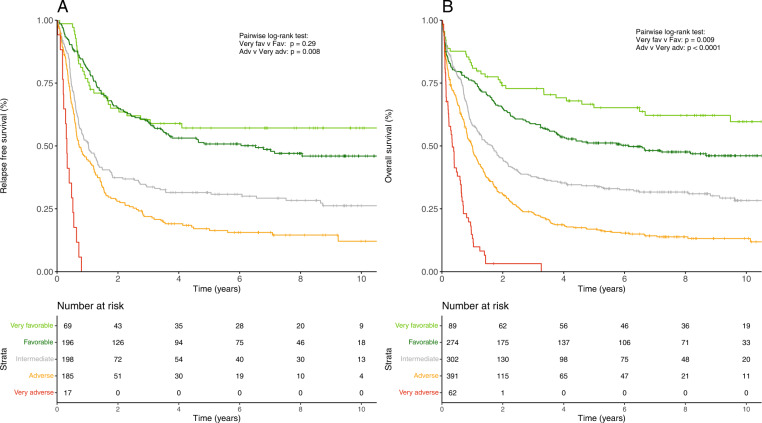
Outcomes of patients according to the proposed refinement of the ELN-2022 classification. **A** Relapse-free survival and **B** overall survival in the entire cohort of 1 118 patients.

In our validation of the ELN-2017 risk classification, we proposed a refinement of the risk stratification system, without introducing additional genetic markers [3]. Within the novel ELN-2022 risk groups, patients with *CBFB::MYH11* or *CEBPA*^bZIP-inf^ mutations still had superior OS to other favorable-risk patients, with an estimated 5-year OS of 71% and 60%, respectively, compared to patients with *RUNX1::RUNX1T1* or *NPM1*^mut^ without *FLT3*^mut^ who achieved 5-year OS rates of 50% and 51%, respectively (Supplementary Fig. 6A, B). On the other hand, patients with complex karyotypes in combination with mutated *TP53* had particularly unfavorable outcomes, with a 5-year RFS and OS of 0% (Supplementary Fig. 6E, F). Based on these observations and previously published data, [23, 26–30] we maintain our proposal to refine the ELN risk groups by delineating a “very favorable” risk group including patients with either *CBFB::MYH11* or *CEBPA*^bZIP-inf^ without cytogenetic changes classified as intermediate or adverse risk (*n* = 89, or 8% of our cohort). On the other hand, patients harboring both a complex karyotype and mutated *TP53* should be considered “very adverse” (*n* = 62; 6% of our cohort). According to this refined classification, CR rates for the very favorable, favorable, intermediate, adverse, and very adverse groups were 76, 72, 67, 47, and 27%, respectively (Supplementary Table 6). RFS and OS for this refined ELN-2022 classification are shown in Fig. 6, and RFS and OS stratified by age in Supplementary Fig. 7. Estimated 5-year OS was 65%, 51%, 34%, 17%, and 0%, respectively. In multivariable analyses adjusting for potential confounders (Supplementary Fig. 8), the “very adverse” group of this refined classification had inferior CR rate, RFS, and OS compared to the adverse group. The very favorable-risk subgroup had longer OS compared with the favorable subgroup, although CR rate and RFS were not significantly different. This OS difference was driven by survival after relapse (Supplementary Fig. 9), which was significantly longer for the very favorable compared with the favorable (*p* = 0.002) and all other subgroups, consistent with reports that patients with *CEBPA* mutations are particularly responsive to salvage therapies [31–34]. Our proposed refinement of the ELN-2022 risk stratification was validated in the AMLSG patient cohort. There, we observed a trend towards better OS of the very favorable compared to the favorable group (5 y OS, 77% vs. 58%; median OS, not reached vs. 8.5 y; *p* = 0.06), and significantly worse survival for the very adverse compared to the adverse group (5 y OS, 0% vs. 24%; median OS, 0.5 y vs. 1.2 y; *p* < 0.0001).

## Discussion

The ELN-2017 recommendations for risk stratification of AML have achieved broad influence in clinical practice and were adopted worldwide [35]. Therefore, it is likely that the changes introduced by the ELN-2022 guidelines will also find their way into clinical trials and routine practice. Because only ~15% of AML patients are reclassified by the new recommendations, and outcomes of individual risk groups as well as overall prognostic accuracy remain largely similar, ELN-2022 represents an incremental change over the previous classification. However, for those patients affected by the proposed changes, it is still of utmost importance to evaluate whether that incremental change is a step towards more accurate risk prediction. Our analyses shed a mixed light on the newly introduced changes.

Like ELN-2017, ELN-2022 is a robust risk stratification system applicable in both younger and older patients who undergo intensive treatment. The association between male sex and adverse genetic risk, which we already observed for ELN-2017, still holds true for the new classifier. While this effect is largely due to sex differences in the frequency of mutations in *ASXL1*, *NPM1*, and *RUNX1*, it is augmented by the introduction of additional MR-related mutations as adverse-risk-defining, as presence of these mutations also associated with male sex. [19, 36–38] These findings are in line with a population-based analysis of U.S. SEER data, where male sex was an independent risk factor for worse OS [39]. However, gender did not associate with OS in a Swedish cohort study [40].

ELN-2022 recognizes MR mutations in *BCOR*, *EZH2*, *SF3B1*, *SRSF2*, *STAG2*, *U2AF1*, and *ZRSR2* as independent markers of adverse risk. The idea that these mutations reflect myelodysplasia is reflected in the WHO-classification and the International-Consensus-Classification which – with the exemption of *RUNX1* in the WHO-classification – also see these mutations as defining AML with myelodysplasia-related genetic changes [41, 42]. While these mutations mostly occur in the setting of sAML, their prognostic significance is not entirely clear [43].

In our cohort, recognition of these mutations as adverse-risk markers lead to reclassification of 79 patients, corresponding to about 7% of the entire cohort and 45% of all reclassified patients. However, our analysis does not support this modification to the risk classification, since RFS and OS of patients re-assigned to the adverse-risk group because of MR mutations were more favorable compared to other adverse risk patients, and not significantly worse than for the remaining intermediate-risk group. While the validation cohort confirms significantly better OS compared to other adverse-risk patients, it also shows worse outcomes compared to intermediate-risk. This outcome might nuance our finding but does not unequivocally support grouping MR-mutated with other adverse-risk patients. In summary, MR mutations in the absence of other, previously recognized adverse-risk markers do not constitute major independent drivers of poor outcomes in younger or elderly patients who receive intensive induction therapy. Hence, these patients might be better classified as intermediate-risk.

The new classification simplifies risk stratification by no longer considering *FLT3*-ITD:wt allelic ratio. This change is supported by our analyses as we did not find significant differences in survival when sub-stratifying the new ELN-2022 risk groups by the presence of *FLT3*-ITD, or by *FLT3*-ITD ratio, despite our cohort being treated in the pre-*FLT3*-inhibitor era. Given the disease-modifying effect of *FLT3* inhibitors, the unfavorable prognostic impact of *FLT3*-ITDs is expected to be reduced further in patients receiving TKI treatment along with frontline intensive therapy [11].

While the ELN risk groups achieve reliable prognostic stratification, further refinement through identification of particularly favorable or adverse subgroups may be clinically beneficial, particularly if this can be achieved without including additional markers. To this end, we [3] and others [23, 26, 27] have reported that *CBFB::MYH11* rearrangement associates with better outcomes than *RUNX1::RUNX1T1*, although a previous study from the UK did not find a difference between these subgroups [44]. In our validation of the ELN-2017 risk groups, we identified patients with biallelic *CEBPA* mutations as another subgroup with particularly favorable outcomes [23, 31]. Meanwhile, it has become clear that *CEBPA*^bZIP-inf^ mutations, rather than biallelic mutations, are the *CEBPA* variants most specifically associated with good outcome [13]. In line with these data, our analysis show that in the context of ELN-2022, patients carrying *CBFB::MYH11* or *CEBPA*^bZIP-inf^ constitute a subset with “very favorable” outcomes that can be separated from the remaining “favorable” patients.

On the other side, patients with both a mutation in *TP53* and a complex karyotype have dismal survival, with a 5-year OS of 0% [3, 28, 29]. Because of this grave unmet clinical need, the apparent lack of benefit from established intensive therapies, and inferior outcomes even compared to other ELN-2022 adverse risk patients, assigning these patients into a distinct “very unfavorable” risk group seems warranted. This group should be treated on clinical trials whenever possible.

The major strength of our study is the large patient cohort which was, across a broad age range, uniformly treated using cytarabine- and anthracycline- based induction regimens. Therefore, we can avoid biases potentially introduced by combining patient cohorts treated on different protocols with varying inclusion and exclusion criteria and treatment approaches. Furthermore, many intermediate- and adverse-risk patients received an allogeneic transplant in first remission, reflecting current standards of care. Limitations of our analysis include the fact that none of the patients in our cohort received novel agents recently introduced into the frontline standard of care, such as gemtuzumab-ozogamicin, midostaurin or CPX-351. These new therapies have been shown to improve outcomes in specific patient subgroups. Our validation study, and arguably the ELN risk stratification itself, do not reflect such subgroup-specific effects of novel, often genetically targeted, therapies.

Importantly, the ELN risk groups were developed based on data from cohorts of relatively young patients who were able to receive intensive induction chemotherapy, usually in the context of clinical trials or registries. While our study included patients fit for intensive therapy with no upper age limit, the median age of our cohort was 58 years – approximately 10 years below the age median of all AML patients. Our results, and again the ELN risk groups per se, should not be generalized to the large group of older AML patients receiving less-intensive treatment. In this context, a recent analysis in patients treated with azacitidine and venetoclax showed that the ELN-2017 risk categories appeared to achieve less clear prognostic separation than among intensively treated patients [45].

One reason for the widespread adoption of the ELN risk groups is their relative simplicity, as risk stratification is largely based on individual genetic alterations, while few gene:gene interactions and no non-genetic factors are considered. More comprehensive scores that also incluse clinical parameters such as performance status have been published, but are used less commonly, in part due to their higher complexity [46]. Machine learning approaches incorporating a broader spectrum of risk factors, and using complex mathematical models to derive more granular risk predictions, have been shown to refine prognostic discrimination and may help address some of the challenges outlined before, but are not yet broadly adopted [47, 48].

While the increasing number of approved therapeutics both in the first-line and relapse setting [11, 49–58] is good news for AML patients and clinicians, it also creates an urgent need to move from prognostic classifications that reflect historical outcomes of one specific therapeutic approach, to predictive models that will allow us to compare expected outcomes for different treatment strategies, and thereby select the most promising option. These models will also need to be able to account for (non-)accessibility of certain therapies due to local approval status or economic constraints, and evolve constantly based on the availability of newer therapies and updated clinical results.

In summary, our validation of the ELN-2022 risk stratification shows that more patients now fall in the adverse risk category, which trends towards having better outcomes than the adverse risk category of the previous ELN-2017 recommendations. Specifically, our data suggest the MR mutations newly classified as adverse-risk markers drive this change and should be more appropriately included in the intermediate-risk category. Further refinement of ELN-2022, especially to emphasize the unmet need of patients with a very poor prognosis, would be feasible by using markers already included in the classifier. Considering additional aspects of disease biology beyond gene mutations and incorporating the effects of new drugs as well as dynamic information along the disease course, can be expected to result in further improvements of AML prognostication.

## Supplementary information

Supplemental Material

## Data Availability

Legal restrictions prohibit us from publicly sharing raw sequencing data, which however can be made available upon reasonable request and permission of the local ethics committee.
