# Supplementary material for: Validation and refinement of the 2022 European LeukemiaNet genetic risk stratification of acute myeloid leukemia
Source: Leukemia. 2023 Apr 11;37(6):1234–44. doi: 10.1038/s41375-023-01884-2 (PMC10244159; doi:10.1038/s41375-023-01884-2)
Supplement: Supplementary file 1 — Supplemental Material [file 41375_2023_1884_MOESM1_ESM.docx]

**Supplemental Appendix to**

**Validation and refinement of the 2022 European LeukemiaNet genetic risk stratification of acute myeloid leukemia**

Rausch et al.

# Supplemental information on patients and methods

## Treatment protocols

## AMLCG-1999 trial

Induction therapy: In the AMLCG-1999 trial^1^ (clinicaltrials.gov identifier NCT00266136; recruitment period, 1999 to 2011; patients included in this analysis were recruited between 1999 and 2004), patients <60 years were randomized to receive double induction with either
one cycle of TAD-9 followed by one cycle of HAM on day 21 (TAD-HAM), or two cycles of HAM 21 days apart (HAM-HAM; TAD-9: thioguanine 100 mg/m² twice daily on days 3-9, cytarabine 100 mg/m²/d continuous infusion on days 1+2 and 100 mg/m² twice daily on days 3-8, and daunorubicin 60 mg/m² on days 3-5; HAM: cytarabine 3 g/m² twice daily on days 1-3 and mitoxantrone 10 mg/m² on days 3-5). From 2002, 10% of patients aged ≤60 years (n=14 patients in this analysis) were randomized to a common standard arm of the German AML intergroup and received 7+3 induction therapy (cytarabine 100 mg/m²/d continuous infusion on days 1-7 and daunorubicin 60 mg/m² on days 3-5).

Postremission treatment: Patients <60 years underwent upfront randomization to undergo consolidation either with one cycle of TAD-9 followed by three years of monthly cytarabine-based maintenance chemotherapy, or a single TAD-9 consolidation course followed by autologous stem cell transplantation (autoSCT) and no maintenance. Per the study protocol, all patients aged <60 years with HLA-matched sibling donors were to be offered allogeneic stem cell transplantation (alloSCT) in first CR, irrespective of cytogenetic or molecular risk factors. Patients ≥60 years were randomized to receive induction therapy with one cycle of either TAD-9 or HAM, followed by a second HAM cycle on day 21 only if ≥5% residual blasts were present in the BM on day 16. All patients ≥60 years were to receive one cycle of TAD-9 consolidation followed by maintenance therapy.^2^

## AMLCG-2008 trial

In the AMLCG-2008 trial^3^ (NCT01382147; recruitment period, 2009 to 2012), patients <60years and fit patients up to the age of 70 (‘younger’ patients) were randomized to receive either double induction chemotherapy with TAD-9 and HAM (21 days apart), or dose-dense induction therapy according to the sHAM regimen (cytarabine 3 g/m² [1 g/m² in patients ≥60 years] twice daily on days 1,2,8 and 9; and mitoxantrone 10 mg/m² on days 3,4,10 and 11). AlloSCT from an HLA-matched related or unrelated donor was the recommended postremission therapy for all younger patients achieving CR except those with favorable genetic features (defined as favorable cytogenetics or cytogenetically normal patients with mutated NPM1 and no FLT3- ITD) and good response to induction chemotherapy (<10% blasts in a bone marrow aspirate obtained on d16 after start of induction therapy). For younger patients without a donor, those unable or unwilling to undergo allotransplantation, and those with a favorable risk profile, postremission therapy consisted of one cycle of TAD-9 for consolidation, followed by 3 years of cytarabine-based maintenance therapy.

Less fit patients aged ≥60 years, and all patients aged ≥70 years, were randomized to receive induction therapy according to the HAM regimen (cytarabine, 1g/m² per dose) followed by a second HAM induction cycle on day 21 only if a bone marrow aspirate on day 16 showed ≥5% blasts, or to dose-dense induction with sHAM (cytarabine, 1g/m² per dose). Postremission therapy in this group consisted of one cycle of TAD-9 for consolidation, followed by 3 years of maintenance therapy.

## AMLSG trials

To validate our findings, we analyzed published genetic and survival data of patients treated on three consecutive clinical trials of the German AMLSG study group (n=1540).^4^ In brief, the AML-HD98A trial included younger patients (18-60 years) who received induction chemotherapy with idarubicin, cytarabine and etoposide (ICE). Allogenic transplantation was offered to patients with adverse cytogenetic risk, intermediate-risk patients received allogeneic transplantation or intensive postremission chemotherapy, and low-risk patients received chemotherapy only. The AML-SG 07-04 trial included younger patients who were randomized to induction with either ICE or ICE plus all-trans retinoic acid (ATRA). In the AML-HD98B trial, patients aged ≥60 years were randomized to receive induction with either ICE or ICE plus ATRA.

For this cohort, information on *FLT3*-ITD-to-wild type allelic ratio is not publicly available. Patients that could not be reliably classified according to the ELN-2017 criteria, mostly due to missing data on *FLT3* allelic ratio, as well as patients with acute promyelocytic leukemia, were excluded, leaving 1178 patients for the validation cohort (median age, 51 years; range, 18-84 years; 83% aged <60 years). Of note, the lack of data on *FLT3*-ITD allelic ratio does not interfere with classifying patients into the proposed “very favorable” and “very adverse” subgroups, allowing us to use this cohort for the purpose of validating our refinement of the ELN-2022 classification.

## Definitions of clinical endpoints

Based on generally accepted recommendations,^5,6^ clinical endpoints were defined as follows: Complete remission (CR) required a bone marrow (BM) aspirate with cellularity greater than 20% and maturation of all cell lines, less than 5% blasts and no Auer rods; and in the peripheral blood, an absolute neutrophil count of ≥1,500/µL, platelet count of ≥100,000/µL, and no leukemic blasts; and no evidence of extramedullary leukemia. Relapse was defined by the presence of ≥5% BM blasts, or Auer rods, or leukemic blasts in the peripheral blood, or the development of extramedullary leukemia. Event-free survival was measured from the day of randomization to the day of treatment failure, hematologic relapse or death from any cause, whichever occurs first. Treatment failure was defined as not achieving CR after two cyles of intensive chemotherapy. Patients evaluable for response but not achieving CR, by the defined landmark and patients who die before the defined landmark without response were considered an event at randomization Patients achieving CR who did not relapse or die were censored on the date they were last assessed for response. Relapse-free survival (RFS) was measured from the date of CR until the date of relapse or death; patients alive and in CR were censored at last follow-up. Overall survival (OS) was measured from the date of study entry until the date of death, and patients alive at last follow-up were censored.

## Analysis of alloSCT as time-dependent covariate

Since a patient can only undergo alloSCT if they have survived until transplant, the usual survival analysis using a Kaplan-Meier plot would overestimate the benefit of alloSCT. This effect is called guarantee-time bias or survivor bias. To counteraxct this effect, we have analyzed alloSCT as a time-dependent covariate. For a review of this method see Fischer LD and Lin DY, Annu. Rev. Public Health. 1999. 20:145-57. In short, every observation of a transplanted patient is split into two, with one observation beginning at the time of diagnosis and being censored at the time of alloSCT, and one observation only starting at the time of transplant. Time dependent covariates are implemented using the tmerge()-function from the R-package survival (version 2.43-3). Based on these data, Simon-Makuch curves are plotted using the survival and survminer R-packages.

# Supplemental Tables

## Supplementary Table 1: Comparison of the AMLCG Cohort and the Validation Cohort

|  | All patients  AMLCG | AMLCG by ELN-2022 risk-group | | | All patients  AML-SG | AML-SG by ELN-2022 risk-group | | |
| --- | --- | --- | --- | --- | --- | --- | --- | --- |
|  |  | Favorable | Intermediate | Adverse |  | Favorable | Intermediate | Adverse |
| Patients, n | 1 118 | 363 | 302 | 453 | 1160 | 457 | 199 | 504 |
| Age, median (range) | 58 (18-86) | 52 (18-86) | 55 (18-83) | 62 (21-80) | 51 (18-84) | 47 (19-79) | 50 (18-84) | 55 (19-79) |
| Male sex | 573 | 166 | 133 | 274 | 635 | 226 | 105 | 304 |
| AML subtype |  |  |  |  |  |  |  |  |
| De-novo AML | 937 | 334 | 258 | 345 | 1018 | 435 | 170 | 413 |
| Secondary AML | 124 | 17 | 27 | 80 | 55 | 6 | 10 | 39 |
| Therapy-related AML | 57 | 12 | 17 | 28 | 59 | 12 | 15 | 32 |
| Type unknown | 0 | 0 | 0 | 0 | 28 | 4 | 4 | 20 |
| WBC at diagnosis [G/l], median (range) | 20.4  (0.1-798) | 24.1  (0.4-798) | 36.5  (0.1-786) | 11.5  (0.5-666) | 11.8  (0.2-533) | 21.4  (0.2-295) | 3.9  (0.4-427) | 8.1 (  0.5-533) |
| Bone marrow blasts [%], median (range) | 80 (6-100) | 80 (6-100) | 83 (10-100) | 71 (9-100) | 70 (0-100 ) | 78 (2-100) | 70 (0-98) | 68 (4-100) |
| ELN-2017 risk group |  |  |  |  |  |  |  |  |
| Favorable | 423 | 351 | 61 | 11 | 446 | 442 | 2 | 2 |
| Intermediate | 295 | 7 | 220 | 68 | 290 | 8 | 195 | 87 |
| Adverse | 400 | 5 | 21 | 374 | 424 | 7 | 2 | 415 |
| Gene Mutations |  |  |  |  |  |  |  |  |
| *ASXL1* | 135 | 14 | 2 | 119 | 60 | 3 | 0 | 57 |
| *BCOR* | 90 | 6 | 3 | 81 | 35 | 6 | 0 | 29 |
| *CEBPA*^bZIP-inf^ | 44 | 44 | 0 | 0 | 66 | 53 | 13 | 0 |
| *CEBPA*^other^ | 39 | 8 | 18 | 13 | 26 | 13 | 4 | 9 |
| *DNMT3A* | 391 | 137 | 157 | 97 | 220 | 138 | 27 | 55 |
| *EZH2* | 44 | 11 | 1 | 32 | 40 | 11 | 0 | 29 |
| *FLT3*-ITD | 303 | 14 | 204 | 85 | 72 | 17 | 2 | 53 |
| *IDH1* | 98 | 43 | 29 | 26 | 81 | 43 | 12 | 26 |
| *IDH2* | 165 | 43 | 48 | 74 | 122 | 46 | 34 | 42 |
| *KMT2A*-PTD^*^ | 56 | 1 | 17 | 38 | 59 | 3 | 18 | 38 |
| *KRAS* | 73 | 26 | 19 | 28 | 60 | 25 | 7 | 28 |
| *NPM1* | 431 | 239 | 164 | 28 | 272 | 261 | 0 | 11 |
| *NRAS* | 216 | 98 | 39 | 79 | 138 | 133 | 24 | 81 |
| *PTPN11* | 114 | 59 | 18 | 37 | 87 | 53 | 7 | 37 |
| *RAD21* | 54 | 27 | 17 | 10 | 30 | 23 | 1 | 6 |
| *RUNX1* | 178 | 7 | 0 | 171 | 126 | 5 | 0 | 121 |
| *TET2* | 192 | 65 | 51 | 76 | 97 | 48 | 8 | 41 |
| *TP53* | 81 | 2 | 4 | 75 | 95 | 3 | 1 | 91 |
| *SF3B1* | 45 | 4 | 1 | 40 | 32 | 0 | 0 | 32 |
| *SRSF2* | 135 | 21 | 1 | 113 | 84 | 14 | 0 | 70 |
| *STAG2* | 98 | 17 | 1 | 80 | 53 | 8 | 0 | 45 |
| *U2AF1* | 38 | 3 | 0 | 35 | 31 | 0 | 0 | 31 |
| *WT1* | 143 | 48 | 43 | 52 | 39 | 22 | 10 | 7 |
| *ZRSR2* | 12 | 2 | 0 | 10 | 12 | 1 | 0 | 11 |

**KMT2A* status unknown for 134

## Supplementary Table 2: Reasons for risk group re-classification

| ELN-2017 risk group | ELN-2022 risk group | n (%) | Reason for reclassification | n (%) |
| --- | --- | --- | --- | --- |
| Favorable | Favorable | 351 (31%) | - | - |
| Favorable | Intermediate | 61 (5%) | Non-bzip bi*CEBPA* | 3 (2%) |
|  |  |  | *NPM1*^mut^ with *FLT3*-ITD^low^ | 58 (34%) |
| Favorable | Adverse | 11 (1%) | Non-bzip bi*CEBPA*, newMR^mut^ | 3 (2%) |
|  |  |  | *NPM1*^mut^ with *FLT3*-ITD^low^ and newMR^mut^ | 8 (5%) |
| Intermediate | Favorable | 7 (<1%) | siC*EBPA*^bzip^* | 7 (4%) |
| Intermediate | Intermediate | 220 (20%) | - | - |
| Intermediate | Adverse | 68 (6%) | newMR^mut^ | 51 (29%) |
|  |  |  | *NPM1*^mut^ with *FLT3*-ITD^high^ and newMR^mut^ | 11 (7%) |
|  |  |  | *NPM1*^wt^ with *FLT3*-ITD^low^ and newMR^mut^ | 6 (3%) |
| Adverse | Favorable | 5 (<1%) | *ASXL1*^mut^ or *RUNX1*^mut^ with si*CEBPA*^bzip^ | 5 (3%) |
| Adverse | Intermediate | 21 (2%) | *NPM1*^wt^ with *FLT3*-ITD^high^ | 20 (12%) |
|  |  |  | *TP53*^mut^ at <10% VAF | 1 (<1%) |
| Adverse | Adverse | 374 (33%) | - | - |

*one of these also is *NPM1*^mut^ with *FLT3*-ITD^high^

**Categories of patients reclassified from ELN-2017 to ELN-2022**: **Non-bzip biCEBPA:** biallelic *CEBPA* mutation outside the basic leucine zipper domain; ***NPM1*^mut^ with *FLT3*-ITD^low^**: Mutation in *NPM1* and internal tandem duplication (ITD) with an allelic ratio <0.5 in *FLT3*; **Non-bzip bi*CEBPA*, newMR^mut^:** biallelic *CEBPA* mutation outside the basic leucine zipper domain (reclassifying the case from favorable to intermediate) together with a MR-mutation newly classified as adverse (*BCOR, EZH2, SF3B1, SRSF2, STAG2, U2AF1*, or *ZRSR2*) (re-reclassifying the case from intermediate to adverse); ***NPM1*^mut^ with *FLT3*-ITD^low^, newMR^mut^:** Mutation in *NPM1* and internal tandem duplication (ITD) with an allelic ratio <0.5 in FLT3 (reclassifying the case from favorable to intermediate) together with a MR-mutation newly classified as adverse (*BCOR, EZH2, SF3B1, SRSF2, STAG2, U2AF1*, or *ZRSR2*) (re-reclassifying the case from intermediate to adverse); **si*CEBPA*^bzip^:** Monoallelic mutation in the basic leucine zipper domain of *CEBPA*; **newMR^mut^:** MR-mutation newly classified as adverse (*BCOR, EZH2, SF3B1, SRSF2, STAG2, U2AF1*, or *ZRSR2*); ***NPM1*^mut^ with *FLT3*-ITD^high^ and newMR^mut^:** Mutation in *NPM1* and internal tandem duplication (ITD) with an allelic ratio >0.5 in *FLT3* together with a MR-mutation newly classified as adverse (*BCOR, EZH2, SF3B1, SRSF2, STAG2, U2AF1*, or *ZRSR2*); ***NPM1*^wt^ with *FLT3*-ITD^low^ and newMR^mut^:** No mutation in *NPM1* and internal tandem duplication (ITD) with an allelic ratio <0.5 in FLT3 together with a MR-mutation newly classified as adverse (*BCOR, EZH2, SF3B1, SRSF2, STAG2, U2AF1*, or *ZRSR2*); ***ASXL1*^mut^ or *RUNX1*^mut^ with si*CEBPA*^bzip^:** Mutation in *ASXL1* and/or *RUNX1* co-occurring with a monoallelic mutation in the basic leucine zipper domain of *CEBPA;* ***NPM1*^wt^ with *FLT3*-ITD^hig^h:** No mutation in *NPM1* and internal tandem duplication (ITD) with an allelic ratio >0.5 in FLT3; ***TP53*^mut^ at <10% VAF:** TP53 mutation with <10% VAF.

## Supplementary Table 3: Outcomes by ELN-2017 risk group

| ELN-2017 risk group | CR rate [%] | p | 5y RFS [%] (95% CI) | p | 5y OS [%] (95% CI) | **p** |
| --- | --- | --- | --- | --- | --- | --- |
| All patients | | | | | | |
| Favorable | 72 | <0.0001 | 53.1 (47.6-59.1) | <0.0001 | 53.9 (49.3-59.0) | <0.0001 |
| Intermediate | 66 |  | 25.9 (20.4-33.0) |  | 30.8 (25.9-36.7) |  |
| Adverse | 41 |  | 12.1 (7.9-18.5) |  | 12.2 (9.3-16.0) |  |
| Patients <60a | | | | | | |
| Favorable | 75 | 0.03 | 62.4 (55.9-69.7) | 0.006 | 63.6 (58.0-69.8) | <0.0001 |
| Intermediate | 66 |  | 36.8 (28.8-46.9) |  | 41.9 (35.0-50.1) |  |
| Adverse | 43 |  | 22.4 (14.5-34.6) |  | 20.1 (14.8-27.4) |  |
| Patients ≥60a | | | | | | |
| Favorable | 68 | 0.0007 | 36.1 (28.0-46.6) | 0.045 | 38.0 (31.1-46.5) | <0.0001 |
| Intermediate | 66 |  | 11,1 (6.0-20.8) |  | 15.9 (10.5-24.0) |  |
| Adverse | 40 |  | 3.7 (1.2-11.9) |  | 6.5 (3.9-10.7) |  |

## Supplementary Table 4: Characteristics of patients <60y who achieved CR after induction therapy

|  | **All patients** | **ELN-2022 risk-group** | | | **p** |
| --- | --- | --- | --- | --- | --- |
|  |  | Favorable | Intermediate | Adverse |  |
| Patients, n | 381 | 175 | 120 | 86 | - |
| Age, median (range) | 45 (18-59) | 44 (18-59) | 44.5 (19-59) | 47 (21-59) | 0.4878 |
| Male sex | 175 | 72 | 56 | 47 | 0.848 |
| **Disease Type** | | | | | |
| De-novo AML | 354 | 167 | 113 | 74 | - |
| Secondary AML | 17 | 5 | 4 | 8 | **0.0316** |
| Therapy-related AML | 10 | 3 | 3 | 4 | 0.2425 |
| WBC at diagnosis [G/l], median (range) | 23.3 (0.8-486) | 24.1 (1.1-316) | 39 (0.9-486) | 9.4 (0.8-255) | 0.103 |
| Bone marrow blasts [%], median (range) | 80 (13-100) | 80 (19-100) | 81.5 (20-100) | 71 (13-100) | 0.185 |
| **ELN -2017 risk group** | | | | | |
| Favorable | 196 | 171 | 22 | 3 | - |
| Intermediate | 113 | 4 | 91 | 18 | - |
| Adverse | 72 | 0 | 7 | 65 | - |
| **Postremission therapy** | | | | | |
| Allo-SCT | 97 | 32 | 36 | 29 | 0.0499 |
| ASCT | 44 | 19 | 14 | 11 | 0.7023 |
| Consolidation Chemotherapy | 239 | 124 | 70 | 46 | 0.0427 |
| **Genetic mutations detected in pretreatment sample** | | | | | |
| *ASXL1* | 15 | 4 | 1 | 10 | 0.0002 |
| *BCOR* | 19 | 3 | 1 | 15 | 0.0011 |
| *CEBPA*^bZIP-inf^ | 21 | 21 | 0 | 0 | 0.0021 |
| *CEBPA*^other^ | 17 | 7 | 9 | 1 | 0.3128 |
| *DNMT3A* | 125 | 56 | 54 | 15 | 0.0004 |
| *EZH2* | 11 | 7 | 0 | 4 | 0.2773 |
| *FLT3*-ITD | 110 | 8 | 84 | 18 | 0.0785 |
| *FLT3*-ITD low | 50 | 6 | 32 | 12 | 0.856 |
| *FLT3*-ITD high | 60 | 2 | 52 | 6 | 0.011 |
| *IDH1* | 31 | 18 | 12 | 1 | 0.0058 |
| *IDH2* | 42 | 13 | 16 | 13 | 0.174 |
| *KMT2A*-PTD | 12 | 1 | 2 | 9 | 0.0004 |
| *KRAS* | 23 | 11 | 5 | 7 | 0.4385 |
| *NPM1* | 178 | 104 | 66 | 8 | <0.0001 |
| *NRAS* | 88 | 51 | 19 | 19 | 0.8849 |
| *PTPN11* | 43 | 32 | 5 | 6 | 0.178 |
| *RAD21* | 22 | 13 | 6 | 3 | 0.4322 |
| *TET2* | 40 | 18 | 14 | 8 | 0.8419 |
| *TP53* | 11 | 1 | 1 | 9 | 0.0267 |
| *SF3B1* | 7 | 1 | 1 | 5 | 0.0076 |
| *SRSF2* | 16 | 3 | 1 | 12 | 0.0185 |
| *STAG2* | 17 | 5 | 0 | 12 | 0.0139 |
| *U2AF1* | 5 | 0 | 0 | 5 | 0.0005 |
| *WT1* | 68 | 27 | 25 | 16 | 0.8731 |
| *ZRSR2* | 1 | 0 | 0 | 1 | 0.0879 |

^*^*KMT2A* PTD status is unknown for 132 patients.

## Supplementary Table 5: Outcomes by genetic subsets of ELN-2022 risk groups

| **ELN-2022 genetic subset** | **n** | **CR [%]** | **p** | **5y RFS [%], (95% CI)** | **p** | **5y OS [%], (95% CI)** | **p** |
| --- | --- | --- | --- | --- | --- | --- | --- |
| **Favorable** | | | | | | | |
| inv(16)/t(16;16) | 45 | 82 | <0.0001 | 62 (48-80) | <0.0001 | 71 (58-85) | <0.0001 |
| t(8;21) | 36 | 58 |  | 52 (37-73) |  | 50 (36-69) |  |
| *CEBPA* bZIPinf | 44 | 73 |  | 62 (44-87) |  | 60 (46-77) |  |
| *NPM1*mut without *FLT3*-ITD | 238 | 74 |  | 49 (42-58) |  | 51 (45-58) |  |
| **Intermediate** | | | | | | | |
| *NPM1*wt without *FLT3*-ITD | 40 | 58 | <0.0001 | 4 (1-30) | <0.0001 | 23 (13-41) | <0.0001 |
| *NPM1*mut with *FLT3*-ITD | 164 | 68 |  | 38 (30-48) |  | 36 (30-45) |  |
| t(9;11) | 22 | 59 |  | 39 (19-77) |  | 21 (9-48) |  |
| Other | 76 | 66 |  | 29 (18-45) |  | 39 (29-52) |  |
| **Adverse** | | | | | | | |
| *ASXL1*mut or *RUNX1*mut | 52 | 50 | <0.0001 | 10 (3-34) | <0.0001 | 14 (7-29) | <0.0001 |
| Mutations in *BCOR*, *EZH2*, *SF3B1*, *SRSF2*, *STAG2*, *U2AF1* or *ZRSR2* | 79 | 65 |  | 25 (16-41) |  | 26 (18-38) |  |
| Complex karyotype, *TP53mut* | 62 | 27 |  | 0 |  | 0 |  |
| Complex karyotype, *TP53wt* | 38 | 42 |  | 19 (7-52) |  | 18 (9-36) |  |
| Multiple adverse characteristics | 162 | 41 |  | 10 (6-21) |  | 10 (6-16) |  |
| Other adverse characteristics | 60 | 42 |  | 27 (14-54) |  | 27 (17-41) |  |

## Supplementary Table 6: Outcomes by refined ELN-2022 risk group

| **ELN-2022 risk group** | **n** | **CR [%]** | **p** | **5y RFS [%], (95% CI)** | **p** | **5y OS [%], (95% CI)** | **p** |
| --- | --- | --- | --- | --- | --- | --- | --- |
| **All patients** | | | | | | | |
| Very Favorable | 89 | 76 | <0.0001 | 57 (46-70) | <0.0001 | 65 (56-76) | <0.0001 |
| Favorable | 274 | 72 |  | 51 (44-58) |  | 51 (46-58) |  |
| Intermediate | 302 | 67 |  | 32 (26-39) |  | 34 (29-40) |  |
| Adverse | 391 | 47 |  | 17 (12-24) |  | 17 (14-21) |  |
| Very Adverse | 62 | 27 |  | 0 |  | 0 |  |
| **Patients <60a** | | | | | | | |
| Very Favorable | 69 | 80 | <0.0001 | 63 (51-77) | <0.0001 | 71 (61-83) | <0.0001 |
| Favorable | 165 | 73 |  | 59 (51-69) |  | 59 (51-67) |  |
| Intermediate | 180 | 67 |  | 41 (33-51) |  | 44 (37-52) |  |
| Adverse | 170 | 48 |  | 31 (22-43) |  | 27 (21-35) |  |
| Very Adverse | 16 | 31 |  | 0 |  | 0 |  |
| **Patients ≥60a** | | | | | | | |
| Very Favorable | 20 | 70 | <0.0001 | 36 (18-72) | 0.01 | 45 (28-73) | <0.0001 |
| Favorable | 109 | 70 |  | 37 (28-50) |  | 39 (31-50) |  |
| Intermediate | 122 | 64 |  | 16 (10-27) |  | 19 (13-28) |  |
| Adverse | 221 | 47 |  | 6 (3-14) |  | 9 (6-14) |  |
| Very Adverse | 46 | 26 |  | 0 |  | 0 |  |

# References

1. Büchner T, Berdel WE, Haferlach C, et al. Age-related risk profile and chemotherapy dose response in acute myeloid leukemia: a study by the German Acute Myeloid Leukemia Cooperative Group. *J Clin Oncol*. 2009;27(1):61-69.

2. Büchner T, Schlenk RF, Schaich M, et al. Acute Myeloid Leukemia (AML): different treatment strategies versus a common standard arm--combined prospective analysis by the German AML Intergroup. *J Clin Oncol*. 2012;30(29):3604-3610.

3. Braess J, Amler S, Kreuzer KA, et al. Sequential high-dose cytarabine and mitoxantrone (S-HAM) versus standard double induction in acute myeloid leukemia-a phase 3 study. *Leukemia*. 2018;32(12):2558-2571.

4. Papaemmanuil E, Gerstung M, Bullinger L, et al. Genomic Classification and Prognosis in Acute Myeloid Leukemia. *N Engl J Med*. 2016;374(23):2209-2221.

5. Cheson BD, Bennett JM, Kopecky KJ, et al. Revised Recommendations of the International Working Group for Diagnosis, Standardization of Response Criteria, Treatment Outcomes, and Reporting Standards for Therapeutic Trials in Acute Myeloid Leukemia. *Journal of Clinical Oncology*. 2003;21(24):4642-4649.

6. Döhner H, Wei AH, Appelbaum FR, et al. Diagnosis and Management of AML in Adults: 2022 ELN Recommendations from an International Expert Panel. *Blood*. 2022.

7. Blanche P, Dartigues JF, Jacqmin-Gadda H. Estimating and comparing time-dependent areas under receiver operating characteristic curves for censored event times with competing risks. *Stat Med*. 2013;32(30):5381-5397.

# Supplementary Figures

## Supplementary Figure 1


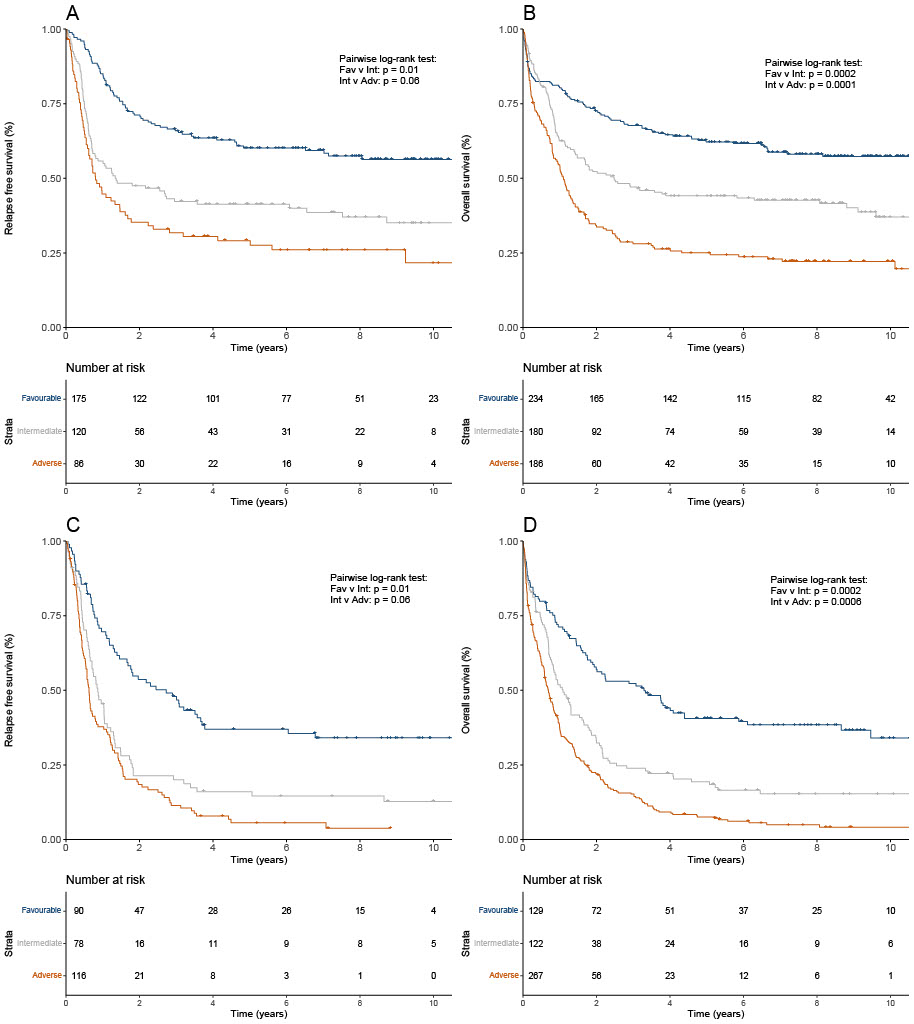


**Outcomes of patients according to the ELN-2022 risk groups, stratified by age group**. A Relapse-free survival and B overall survival according to ELN-2022 risk group in 600 patients <60y. C Relapse-free survival and D overall survival according to ELN-2022 risk group in 518 patients ≥60y.

## Supplementary Figure 2


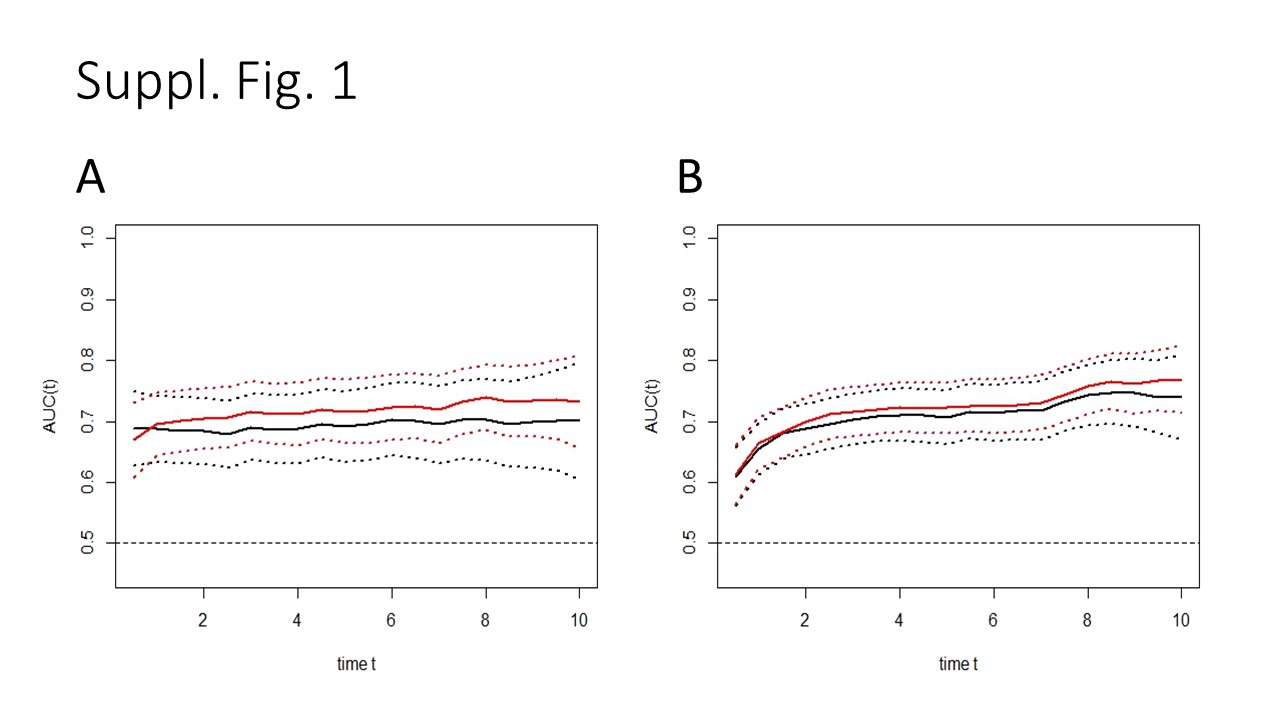


Time-dependent areas under the receiver operating characteristic curves (AUC) for the association between ELN-2017 risk groups, ELN-2022 risk groups and outcomes calculated using the timeROC R-package.^7^ For every 6-month interval from diagnosis to 10y time dependent AUCs are calculated for the association of the risk groups with A RFS and B OS. At each time point, the marker with the higher AUC performs better in correctly predicting the survival outcomes of individual patients at that time. For RFS, ELN-2017 performs significantly better at 2.5y (p=0.017), 3y (y=0.033), and 3.5 y (p=0.041). For OS, ELN-2017 performs significantly better at 9.5y (p=0.049). Association to ELN-2017 risk groups is shown in red, while association to ELN-2022 risk groups are shown in black. Dashed lines represent the respective confidence intervals.

## Supplementary Figure 3


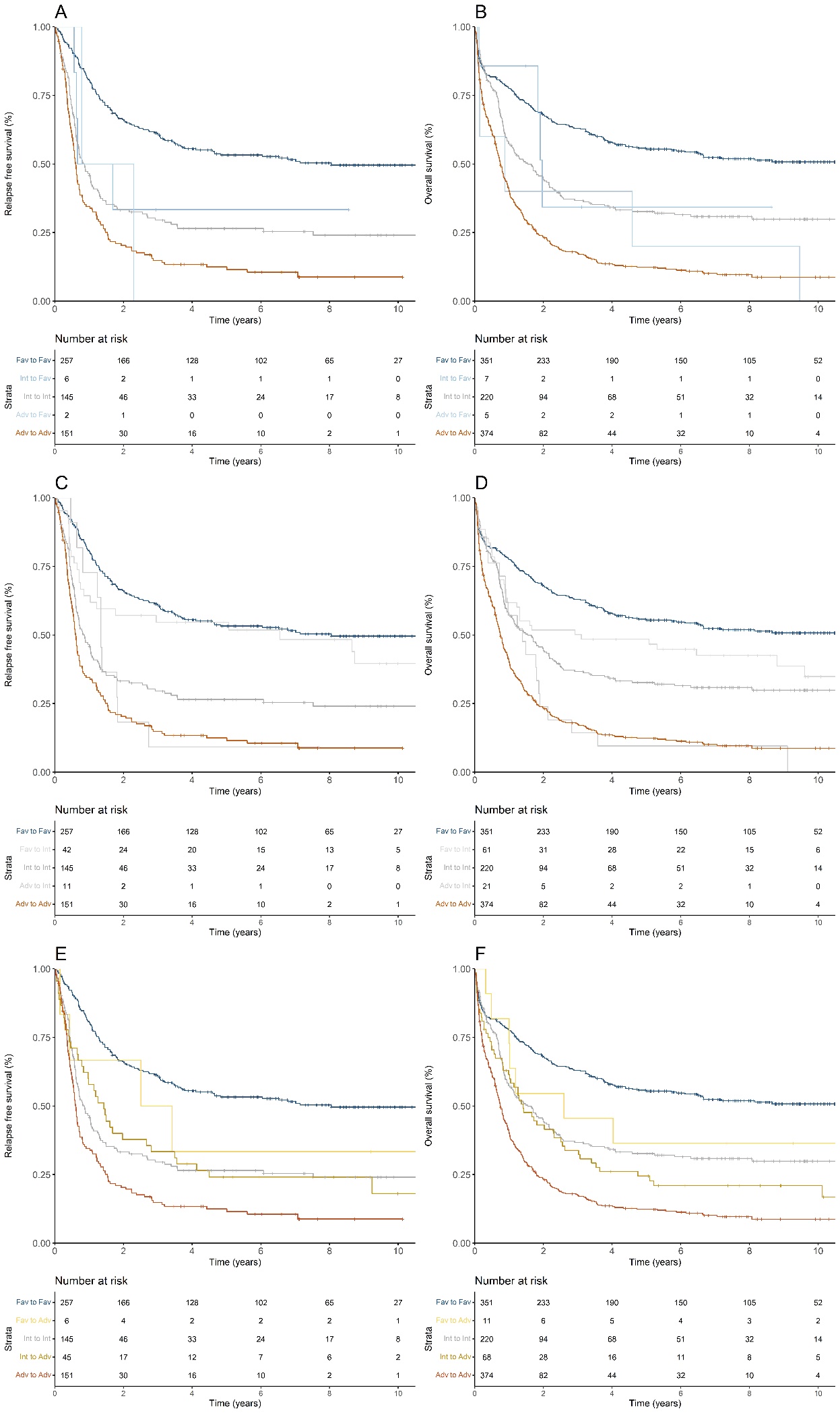


Outcomes of patients newly classified into a risk-group by ELN-2022 compared to those classified equally in both classifications. A RFS and B OS for those newly classified as favorable. C RFS and D OS for those newly classified as intermediate. E RFS and F OS for those newly classified as adverse.

## Supplementary Figure 4


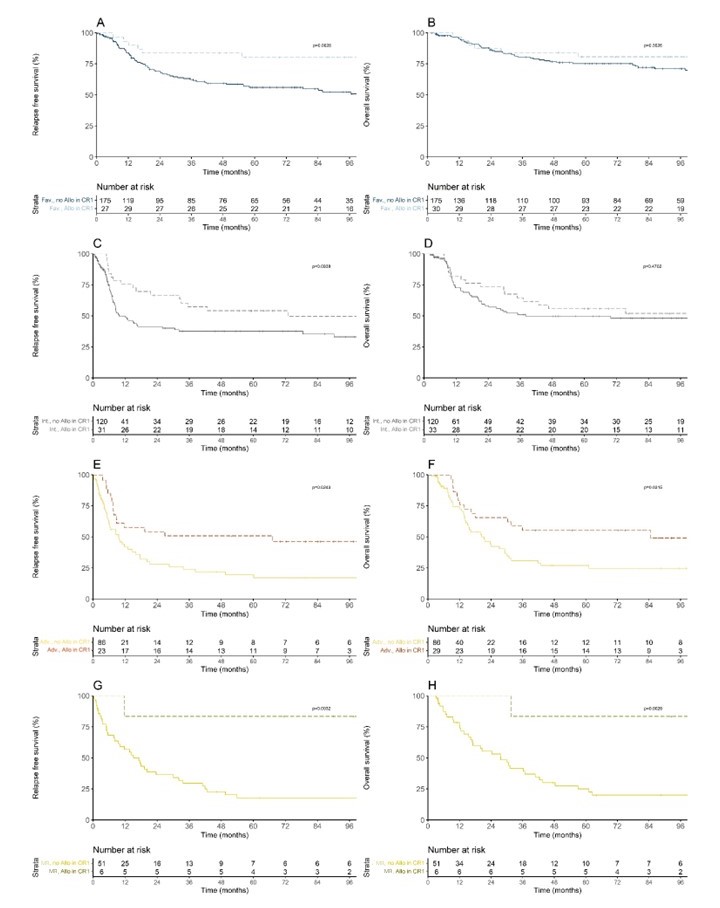


**Outcomes stratified by postremission treatment received in CR1.** Simon-Makuch plots of RFS (A, C, E, G) and OS (B, D, F, H) of patients in the ELN-2022 favorable (A, B), intermediate (C, D) and adverse (E, F) risk groups, as well as those reclassified to adverse based on the presence of a MR-mutation (G, H), who reached CR after induction therapy and were <60y old. Dashed lines show allotransplanted patients, solid lines represent consolidation therapy with autologous SCT or chemotherapy only. RFS was improved by allogeneic transplantation for favorable (p=0.003) and adverse-risk (p=0.02) patients. OS was significantly improved only for those in the adverse risk-group (p=0.03). Patients reclassified to adverse risk based on the presence of a MR mutation seemed to profit strongly from allogeneic transplantation in CR1 (p=0.0032 for RFS and p=0.0026 for OS).

## Supplementary Figure 5


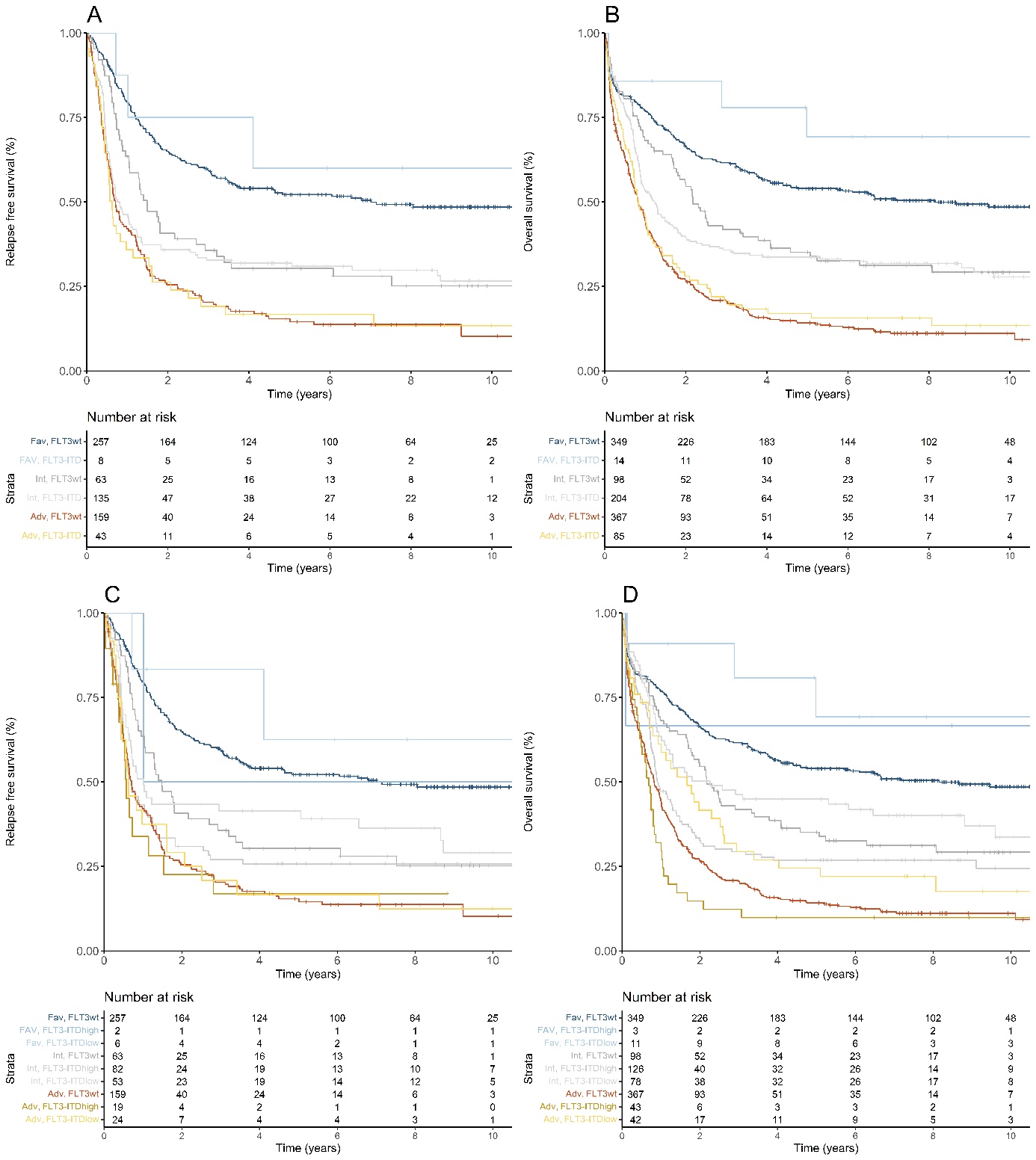


**Outcomes of patients with *FLT3*-ITD according to the ELN-2022 risk groups.** A RFS and B OS of all 1,118 patients stratified by ELN-2022 risk group and the presence of a *FLT3*-ITD. Within each risk group, no significant difference in outcome is observed. C RFS and D OS of all 1,118 patients stratified by ELN-2022 risk group and the presence of a *FLT3*-ITD at a high or low allelic ratio. Within the adverse risk group, those with *FLT3*-ITD^low^ do significantly better than those with *FLT3*-ITD^high^.

## Supplementary Figure 6


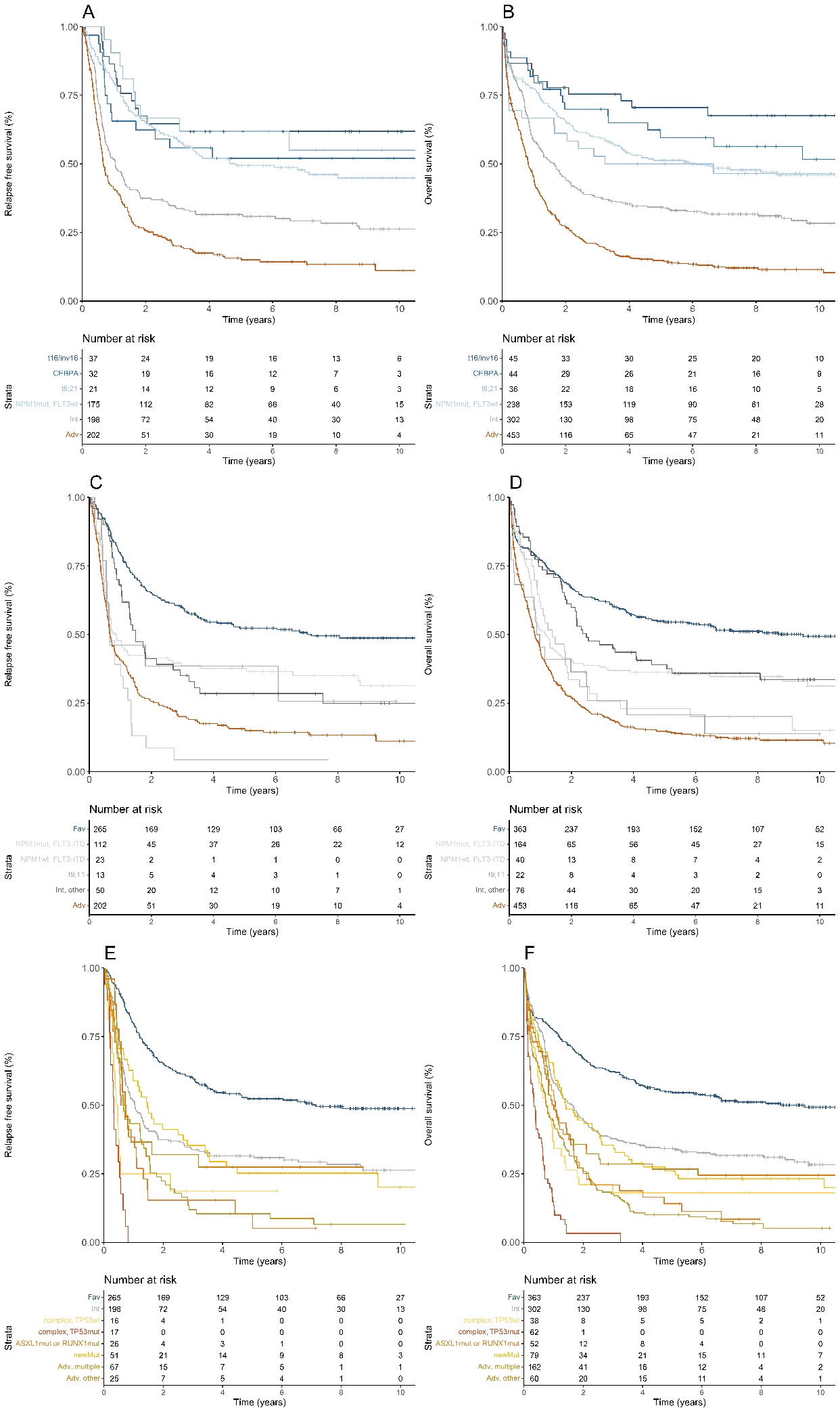


**Outcomes of genetic subsets within the ELN-2022 risk groups.** A RFS and B OS of the ELN favorable risk group, substratified by the marker that warrants inclusion of a patient in the group compared to the intermediate and adverse groups. C RFS and D OS of the ELN intermediate risk group, substratified by the marker that warrants inclusion of a patient in the group compared to the favorable and adverse groups. E RFS and F OS of the ELN adverse risk group, substratified by markers or combinations of markers that warrant inclusion of a patient in the group compared to the favorable and intermediate groups.

## Supplementary Figure 7


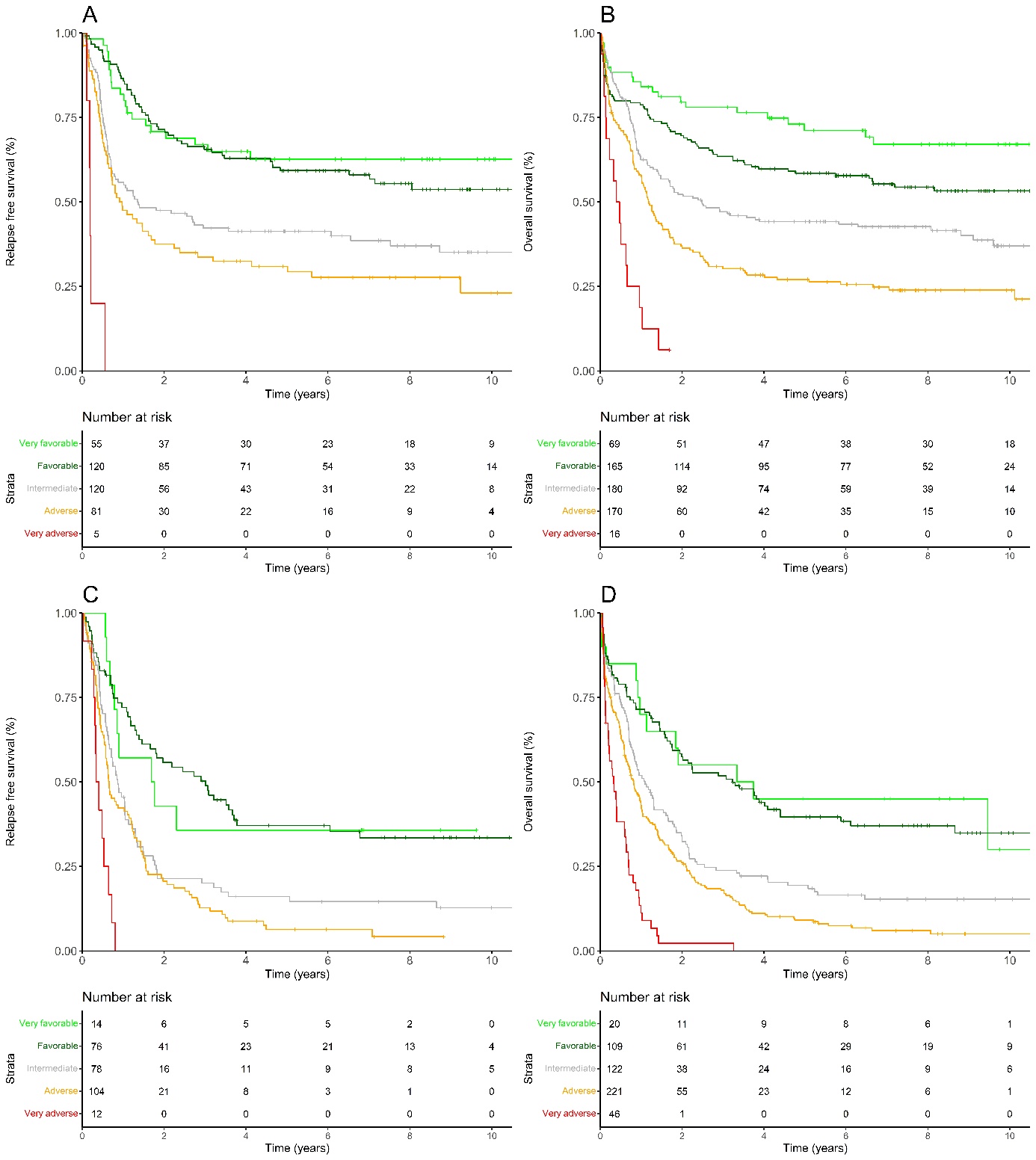


Pairwise log-rank test:

Very fav v Fav: p = 0.89

Adv v Very adv: p = 0.02

Pairwise log-rank test:

Very fav v Fav: p = 0.71

Adv v Very adv: p = 0.005

Pairwise log-rank test:

Very fav v Fav: p = 0.5

Adv v Very adv: p = 0.047

Pairwise log-rank test:

Very fav v Fav: p = 0.046

Adv v Very adv: p = 0.0002

**Outcomes of patients according to the proposed refinement of the ELN-2022 risk groups, stratified by age group.** A Relapse-free survival and B overall survival according to refined ELN-2022 risk group in 600 patients <60y. C Relapse-free survival and D overall survival according to refined ELN-2022 risk group in 518 patients ≥60y.

## Supplementary Figure 8


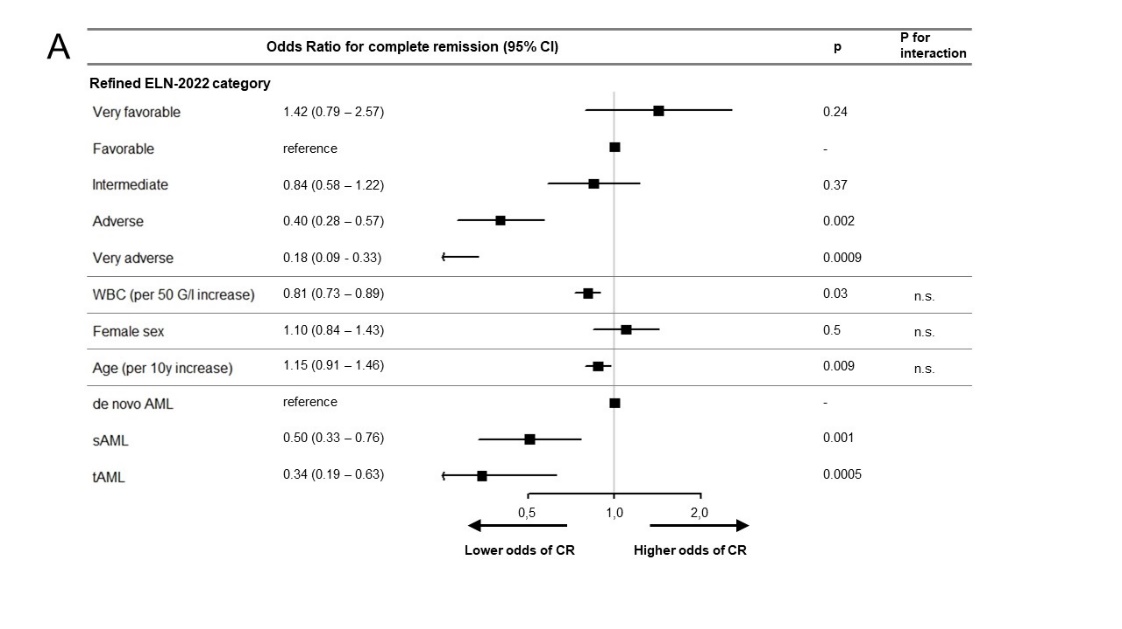

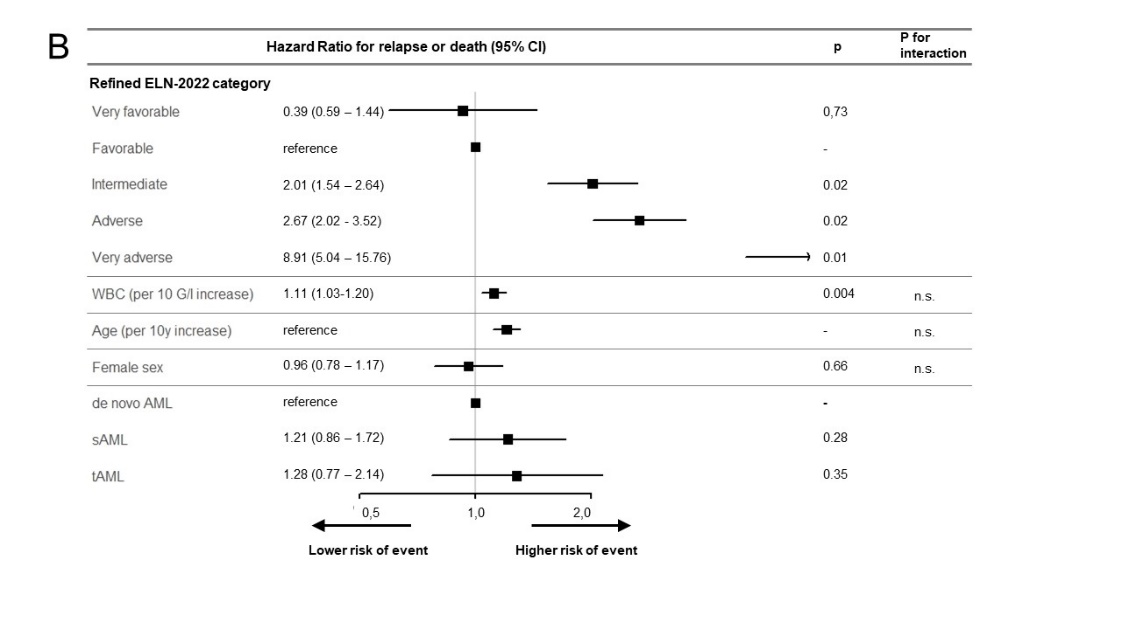

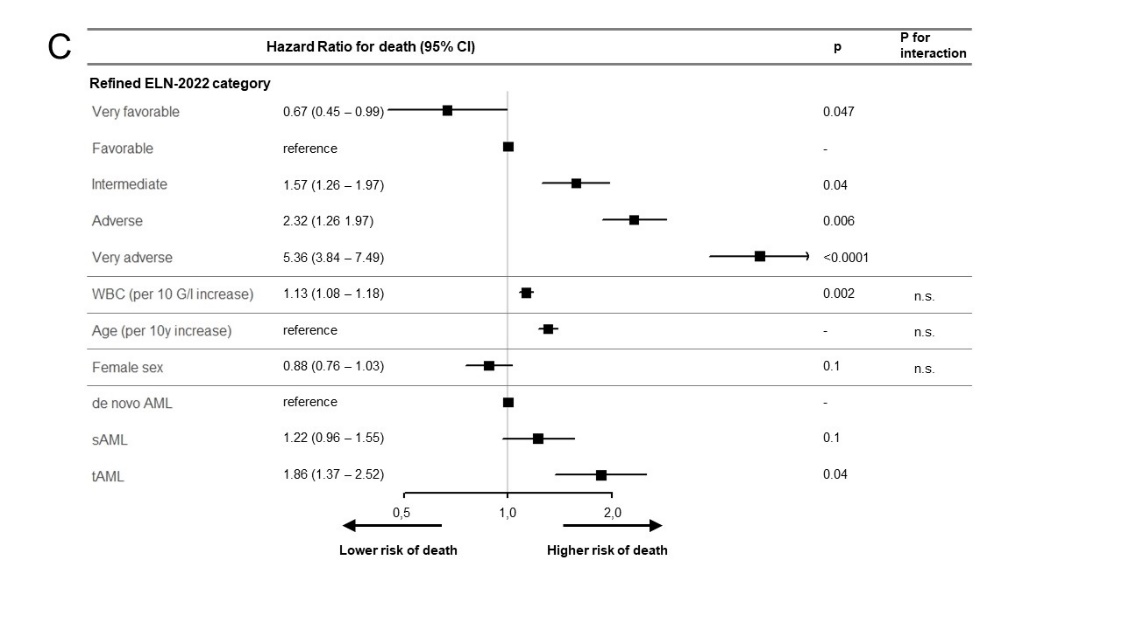


Multivariate analyses of outcomes according to the refined ELN-2022 genetic risk groups and further pretreatment prognostic variables. A Forest plot showing odds ratios from a logistic regression model for achievement of complete remission. B Forest plot showing hazard ratios from a Cox proportional hazards model for relapse-free survival. C Forest plot showing hazard ratios from a Cox proportional hazards model for overall survival. Interaction P values refer to an interaction between the ELN-2022 risk groups and the respective variable.

## Supplementary Figure 9


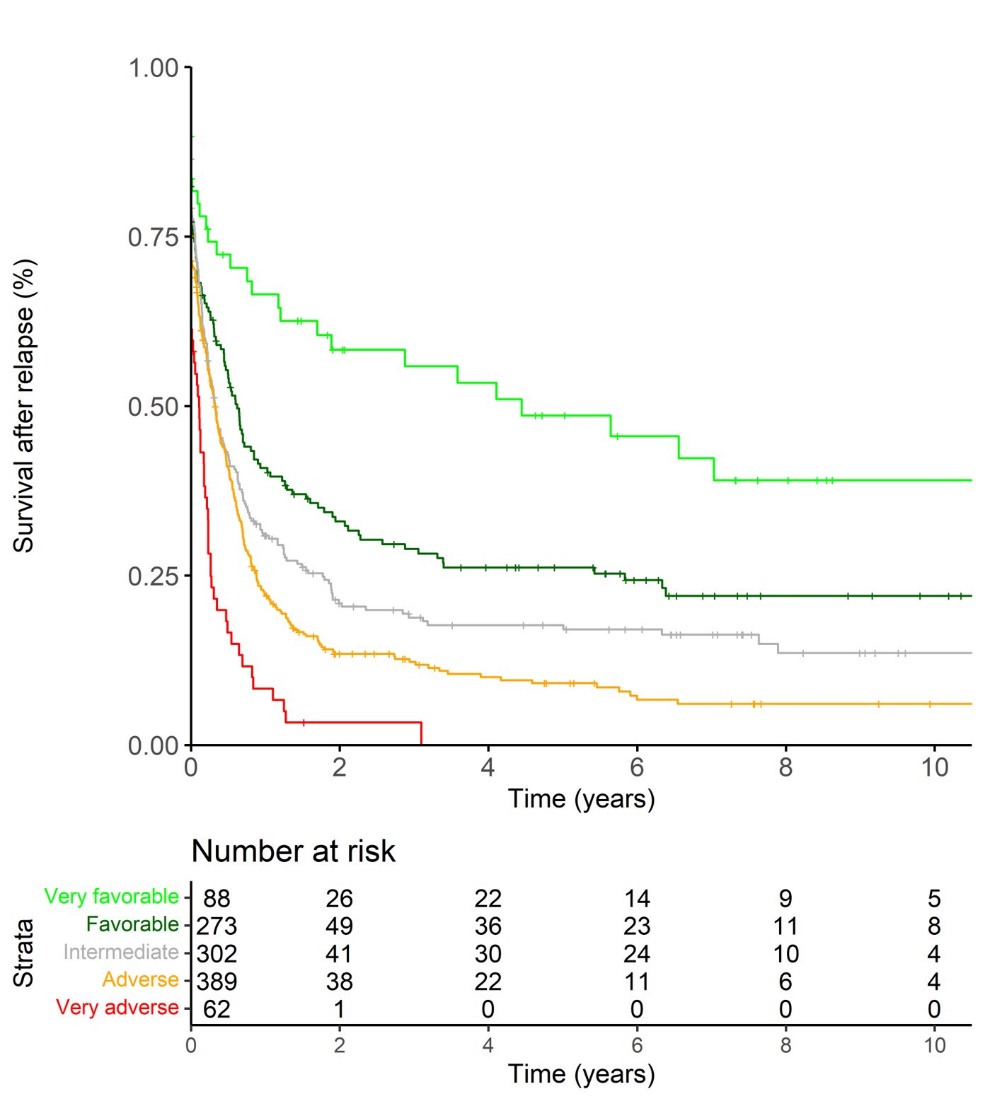


Pairwise log-rank test:

Very fav v Fav: p = 0.002

Survival after relapse of patients according to the proposed refinement of the ELN-2022 risk groups, stratified by age group.
